# CD70 CAR-T cells empowered by TS-2021 through ex vivo transduction show potent antitumor efficacy against glioblastoma

**DOI:** 10.1186/s13046-025-03431-6

**Published:** 2025-06-05

**Authors:** Sheng Fang, Jiankun Wu, Yida Liu, Peiwen Wang, Guiqiang Yuan, Jiajia Gao, Wenxin Zhang, Junwen Zhang, Fusheng Liu

**Affiliations:** 1 Brain Tumor Research Center, Beijing Neurosurgical Institute, Capital Medical University, Beijing, 100070 China; 2 Department of Neurosurgery, Beijing Tiantan Hospital Affiliated to Capital Medical University, Beijing, 100070 China; 3 Beijing Laboratory of Biomedical Materials, Beijing, 100070 China

**Keywords:** Glioblastoma, CAR-T-cell immunotherapy, Oncolytic adenovirus, CD70, IL15

## Abstract

**Background:**

Chimeric antigen receptor (CAR) T-cell therapy has shown limited efficacy in glioblastoma (GBM) due to tumor antigen heterogeneity and the immunosuppressive microenvironment. To address these barriers, we developed a novel combinatorial approach: engineering CAR-T cells with third-generation oncolytic adenoviruses (OAd) to enable targeted viral delivery and sustained immune activation. Unlike conventional OAd administration, this strategy leverages CAR-T cells as tumor-tropic vectors for localized oncolysis and cytokine modulation.

**Methods:**

CD70-specific CAR-T cells were transduced with two third-generation OAds (E1B19K/E3-deleted, replication-selective): OAd-GFP (control) or OAd-IL15 (TS-2021), generating CAR-T^OAd−GFP^ and CAR-T^TS−2021^. Viral replication kinetics and CAR-T expansion were assessed in vitro. OAd delivery efficiency was quantified by co-culturing CAR-T^OAd^ cells with GBM cells. Flow cytometry was used to analyze IL15-mediated effects on stem-like markers (CCR7, CD45RA) and exhaustion markers (PD-1, TIM-3, and LAG-3) after repeated antigen stimulation. Antitumor activity was evaluated in vitro using cytotoxicity assays and in NCG mice bearing orthotopic GBM xenografts. Mechanistic studies were conducted using RNA-seq and Western blotting.

**Results:**

In this study, we found that genetically engineered OAd-GFP can specifically replicate within CAR-T cells and be precisely delivered to GBM through an antigen-specific mechanism. Prolonged antigen stimulation induced T-cell exhaustion, limiting the efficacy of CAR-T therapy. TS-2021-infected CAR-T cells exhibited enhanced expansion and persistence in vitro, with reduced expression of exhaustion markers under sustained antigen stimulation. IL15 autocrine signaling activated JAK-STAT and MAPK-ERK pathways. This process repaired the DNA damage induced by OAd in CAR-T cells and maintained their expansion and persistence. By combining OAd-mediated oncolysis with IL15-driven CAR-T persistence, CAR-T^TS−2021^ cells demonstrated potent antitumor efficacy against GBM both in vitro and in vivo.

**Conclusions:**

By integrating IL15-armed OAd into CAR-T cells, we demonstrate a synergistic strategy that simultaneously enhances viral oncolysis, sustains T-cell persistence, and counteracts GBM immunosuppression. This approach addresses both antigenic heterogeneity and microenvironment-driven resistance, providing a translatable paradigm for solid tumor immunotherapy.

**Supplementary Information:**

The online version contains supplementary material available at 10.1186/s13046-025-03431-6.

## Background

Glioblastoma (GBM) is the most common and aggressive malignant primary brain tumor in adults [1]. Conventional strategies for treating GBM, including surgery, radiotherapy, and chemotherapy, have limited efficacy, with a median overall survival of no more than 15 months [2, 3]. Among several novel therapeutic strategies investigated, chimeric antigen receptor (CAR) T-cell immunotherapy has received widespread attention [4–6]. Although CAR-T immunotherapy is effective in treating hematological neoplasms, GBM treatment faces multiple challenges [7, 8]. The initial efficacy (tumor regression) of CAR-T-cell immunotherapy is limited by inadequate tumor infiltration, a tumor-associated immunosuppressive environment, and T-cell exhaustion [9–11]. The heterogeneity of target antigens, both among individuals and within tumors, is the greatest obstacle to the success of CAR-T-cell immunotherapy in GBM [12, 13]. Antigen mutations or loss (AML) frequently cause tumor relapse following regression, contributing to acquired resistance in GBM [14–16]. Thus, innovative strategies need to be developed to address the resistance to CAR-T-cell immunotherapy in GBM.

Oncolytic virus therapy is a nascent and promising strategy for treating GBM. In this technique, oncolytic viruses are used to selectively destroy tumor cells while activating the host’s antitumor immune response [17]. Incorporating immunostimulatory factors can counteract tumor immune evasion and amplify antitumor immunity [18–21]. However, oncolytic viruses cannot identify tumor cells. Intravenous administration often results in rapid viral clearance, while the blood-brain barrier further restricts the delivery of viruses to the tumor site [22, 23]. Consequently, in clinical trials, most oncolytic viruses used for treating malignant gliomas are delivered through intratumoral injection [24–27]. The immunosuppressive microenvironment of GBM presents considerable challenges to the antitumor immune response triggered by tumor antigens released after oncolytic virus-mediated tumor cell lysis [28]. The integration of oncolytic virotherapy and CAR-T-cell immunotherapy to improve the efficacy against GBM is under investigation.

The third-generation oncolytic adenovirus (OAd) is genetically engineered based on Ki67 and transforming growth factor-β2 (TGF-β2). Viral replication is driven by the Ki67 promoter and the TGF-β2 5′UTR, both of which are closely associated with the malignancy of glioma, ensuring higher tumor specificity and safety. The OAd vector, which contains the IL15 gene (designated as TS-2021), further enhances antitumor immunity by regulating the replication and release of IL15 [29]. TS-2021 has shown potential therapeutic efficacy in clinical trials for recurrent GBM.

A member of the tumor necrosis factor (TNF) superfamily known as CD70 is a promising target for GBM [30, 31]. The application of CAR-T cells targeting CD70 is a promising strategy for GBM treatment [31–34]. In this study, a CD70 CAR was designed and constructed based on previous study [35] and successfully introduced into T cells. We assessed the potential of CAR-T cells as carriers for delivering OAd by pre-infecting T/CAR-T cells in vitro with OAd encoding the EGFP gene (OAd-GFP) using a spin infection protocol (T^OAd−GFP^ and CAR-T^OAd−GFP^). CAR-T^OAd−GFP^ cells efficiently delivered OAd-GFP to GBM cells via an antigen-specific mechanism, significantly increasing the anti-GBM efficacy of CAR-T cells in vitro and in vivo. However, sustained antigen stimulation induced T-cell dysfunction, hindering the efficacy of CAR-T therapy. TS-2021-infected CAR-T (CAR-T^TS−2021^) cells secreted IL15 via an autocrine mechanism, alleviating T-cell exhaustion, repairing OAd-induced DNA damage, promoting CAR-T-cell proliferation, and sustaining long-term functionality. CAR-T^TS−2021^ cells exhibited robust antitumor efficacy in vitro and in vivo, thus mitigating acquired resistance in GBM. Our findings highlighted the ability of CAR-T^TS−2021^ to overcome resistance to CAR-T immunotherapy in GBM.

## Materials and methods

### Cell lines and culture

The U251 cell line was obtained from the Cell Center, Chinese Academy of Medical Sciences (Beijing, China), the 293T cell line was obtained from ATCC, and the human astrocytes (HA) were obtained from ScienCell Research Laboratories. BT325 and BT01 are primary GBM cells that were developed at the Beijing Neurosurgical Institute [36, 37]. The U251-Luc, BT325-Luc, and BT01-Luc cell lines were established through transduction with lentiviral vectors encoding the luciferase gene (Luc). GBM cells and HEK293T cells were maintained in DMEM (Gibco, USA) supplemented with 10% fetal bovine serum (Gibco, USA) and 1% penicillin/streptomycin (Gibco, USA). HA were cultivated in Astrocyte Medium (AM, 1801, ScienCell). The cell lines used in this study were mycoplasma-free.

### CAR vector construction

The CD70 CAR (consisting of the CD8 signal peptide, trCD27, CD8 hinge & transmembrane domain, 4-1BB, CD3ζ, and EGFP) was engineered following a published method [35], with the CD8 signal peptide inserted before trCD27 and an EGFP reporter used after the self-cleaving peptide sequence. The Mock CAR was designed using the above method. Then, the CAR sequences were synthesized and inserted into a second-generation lentiviral vector (GeneChem Co., Ltd., China) under the control of the human EF1a promoter.

### Lentivirus production

First, 293T cells were expanded in DMEM supplemented with 10% fetal bovine serum. A recombinant lentivirus was prepared through plasmid transfection (CAR vector plasmid, pMD2. G, and psPAX2) into 293T cells. The supernatant was collected 72 h post-transfection, and the lentivirus was filtered through sterile Millex filters (Millipore), concentrated with Amicon Ultra filters (Millipore), and preserved at − 80 °C.

Isolation of human T cells and generation of CAR-T cells.

Human peripheral blood mononuclear cells (PBMCs) were isolated from the blood of healthy donors using a lymphocyte separation medium (LBS1077, Precision BioMedicals, China). Human CD3^+^ T cells were isolated and purified from PBMCs via immunomagnetic negative selection (17951, STEMCELL) and then activated for 48 h with the soluble ImmunoCult™ Human CD3/CD28/CD2 T-Cell Activator (STEMCELL) and 200 U/mL IL2. Activated T cells were transfected with CD70 or Mock CAR-encoding lentivirus (MOI = 10:1) and 5 µg/mL polybrene, followed by centrifugation at 2100 rpm for 90 min at room temperature. The cells were cultured in X-vivo 15 medium (Lonza) supplemented with 200 U/mL IL2 for six days. The CAR-positive rate exceeded 50%.

### Third-generation oad

The construction of OAd-GFP (wild-type human adenovirus type 5 genome lacking the E1B19K and E3 regions, with the human Ki67 promoter linked to the human TGF-β2 5′UTR replacing the E1A promoter and the EGFP gene inserted at the E3 region) and TS-2021 (wild-type human adenovirus type 5 genome with deletions in the E1B19K and E3 regions, the human Ki67 promoter linked to the human TGF-β2 5′UTR replacing the E1A promoter, and the human IL15 gene replacing the EGFP) was described previously [29].

### Infection of T/CAR-T cells with oad

First, OAd was added to T/CAR-T cells at an MOI of 400:1 in the presence of 1× HitransG P (GeneChem Co., Ltd., China), followed by centrifugation at 2100 rpm for 90 min at room temperature. T/CAR-T cells were examined 48 h after infection with OAd-GFP via fluorescence microscopy. Infection rates were assessed at 48 and 144 h post-infection via flow cytometry (FCM).

To evaluate the infectivity of OAd-GFP, OAd-GFP (1.2 × 10^7^VP), CD70 CAR-T^OAd−GFP^ (2 × 10^4^), or Mock CAR-T^OAd−GFP^ (2 × 10^4^) was added to U251 cells (4 × 10^4^) or HA (4 × 10^4^). The fluorescence intensity of GFP was measured using an Axio Observer inverted microscope (ZEISS, Germany), and the data were analyzed using the ImageJ software (NIH, USA).

### Proliferation assay and Trypan blue exclusion test

T/CAR-T cells (5 × 10^4^) were plated in 12-well plates containing X-vivo 15 medium (Lonza) enriched with 200 U/mL IL2 after OAd was added. These T/CAR-T cells were counted every day. To test the viability of T/CAR-T-cells, the above cells were treated with 0.4% trypan blue for 3 min at room temperature and then analyzed using a hemocytometer. The number of stained cells and the total number of cells were calculated to determine the cell viability.

### Measurement of oad titers

CAR-T^OAd−GFP^ and CAR-T^TS−2021^ cells were lysed by two freeze-thaw cycles, and the resulting lysates containing OAd were collected. Viral DNA was separated from the cell lysates using the TIANamp Virus DNA/RNA Kit (Tiangen Biotech, Beijing, China). The virus titer was measured following the method described in another study [38], and the E1A cycle threshold (Ct) in the viral DNA was quantified via E1A-specific quantitative polymerase chain reaction (qPCR). The viral titer was quantified by applying the Ct values to the E1A standard curve (Fig. s8) established using OAd (5.0 × 10^11^VP/mL). The E1A sequences of primers used were as follows: 5′-AACCAGTTGCCGTGAGAGTTG-3′ (forward) and 5′-TCGTTAAGCAAGTCCTCCTCGATACAT-3′ (reverse).

### In vitro cytotoxic assays

Cytotoxicity was evaluated by co-culturing CAR-T/CAR-T^OAd^ cells with GBM-Luc target cells (2 × 10^4^/well) at an E: T ratio of 1:1 in 24-well plates. GBM cells (2 × 10^4^/well) were treated with OAd-GFP or TS-2021 (MOI = 300:1). Next, 2 µL of 15 mg/mL D-luciferin (LABLEAD, China) was added to each well after 24, 48, and 72 h of treatment. Bioluminescence was measured using the IVIS Spectrum system (PerkinElmer) and analyzed using Living Image 4.4 (PerkinElmer) to assess cytotoxicity.

In some studies, to investigate the cytotoxicity mediated by CAR-T cells under cytokine stimulation, IL2 (1.2 ng/mL, STEMCELL), IL7 (0.1 ng/mL, STEMCELL), IL12 (2 ng/mL, STEMCELL), and IL15 (0.2 ng/mL, STEMCELL) were added to the culture medium at their effective dose 50 (ED50) concentrations while co-culturing CAR-T cells with target cells.

### Flow cytometry

Flow cytometry analysis was conducted using a BD Accuri C6 Plus instrument (BD Biosciences). The positive expression rate of CAR and the viral infection rate in T cells were determined by detecting EGFP fluorescence signals. Fluorochrome-conjugated antibodies were used for staining following the manufacturer’s protocol. The antibodies used are detailed in Supplemental Table 1.

### Multiplex cytokine profiling

CAR-T, CAR-T^OAd−GFP^, and CAR-T^TS−2021^ cells were incubated with U251 cells (10^5^/well) in 12-well plates (E: T = 1:1) for 48 h, and the cell-free supernatant collected was analyzed using a Human Cytokine Array Q2000 kit (QAH-CAA-2000, RayBiotech) following the manufacturer’s instructions.

### ELISA

After treating GBM cells (10^5^ cells per well in six-well plates) with OAd at an MOI of 300:1 for 72 h, the supernatants were collected. Next, CAR-T cells (5 × 10^4^) were plated in 12-well plates containing X-vivo 15 medium (Lonza) enriched with 200 U/mL IL2 after adding OAd, and the cell-free supernatants were harvested at 48 h, 96 h, and 144 h. The level of IL15 secretion was quantified in the supernatants collected from GBM cells or CAR-T cells using a human IL15 ELISA kit (EHC013, NeoBioscience) following the manufacturer’s guidelines. After OAd was added, CAR-T cells (5 × 10^4^) were plated in 12-well plates containing X-vivo 15 medium (Lonza) without IL2. The supernatants were collected after 72 h, and IL2 and IL15 secretions were measured using a human IL2 ELISA kit (EHC003, NeoBioscience) and the aforementioned human IL15 ELISA kit following the manufacturer’s instructions.

### Generation of CD70 knockout (KO) cell lines

Two sgRNAs were designed that target a common sequence present in all CD70 transcripts to delete a large fragment of the CD70 coding sequence (CDS). The sgRNA sequences used were as follows: KO#1: 5′-CAGCAGGCTGATGCTACGGG-3′ and KO#2: 5′-CCATGTAGATGCCATCACGA-3′. A lentiviral vector (pLenti-Cas9-CD70 KO) was constructed, and the lentivirus was produced by Shanghai GeneChem Co., Ltd. A flow cytometry assay was conducted to analyze CD70 expression in BT325 and U251 cells seven days after transduction with the CD70 KO-encoding lentivirus.

### RNA sequencing

CAR-T, CAR-T^OAd−GFP^, and CAR-T^TS−2021^ cells were collected three days after adding OAd. Total RNA was extracted, followed by PCR amplification, cDNA library preparation, and subsequent bioinformatics analysis. These processes were conducted by Suzhou GENEWIZ Biotech Co., Ltd.

### Quantitative PCR (qPCR)

Total RNA was extracted from HA, GBM, and CAR-T cells using Total RNA Isolation Reagent (BS258A, Biosharp, China) following the manufacturer’s guidelines. Next, cDNA was synthesized using All-in-One First-Strand Synthesis MasterMix (F0202, LABLEAD, China). Gene expression was quantified using specific primers and analyzed via SYBR qPCR (Applied Biosystems), with β-actin as the internal control. The sequences of primers used were as follows: 5′-CTGGCTGCTGACCGAGG-3′ (β-actin-F), 5′-GAAGGTCTCAAACATGATCTGGGT-3′ (β-actin-R), 5′-CCATATGCCTGTGGAGTGGAA-3′ (Ki67-F), and 5′-CCACCCTTAGCGTGCTCTTGA-3′ (Ki67-R).

### Western blotting analysis

Protein samples were isolated from cells using RIPA lysis buffer (R0020, Solarbio, China) containing a protease inhibitor (Roche) and a phosphatase inhibitor (Roche). The cell lysates were then centrifuged at 10,000×*g* at 4 °C, and the supernatants were collected to determine the protein concentration using the bicinchoninic acid (BCA) method. Proteins were loaded onto gels, separated by SDS-PAGE, and then transferred to polyvinylidene fluoride membranes. The membranes were blocked at room temperature with TBST (Tris-buffered saline, 0.1% Tween 20) containing 5% non-fat dry milk for 1 h. After the membranes were blocked, they were incubated overnight with primary antibodies at 4 °C, washed with TBST, and incubated with secondary antibodies at room temperature for 60 min. The protein bands were visualized using an enhanced chemiluminescence kit (NCM Biotech, China) and imaged using a ChemiDoc MP system (Bio-Rad, USA). The antibodies used are detailed in Supplemental Table 1.

### In vivo experiments

The NCG mice were obtained from GemPharmatech Co., Ltd. and housed in a specific pathogen-free environment. Under isoflurane anesthesia, GBM cells were stereotaxically injected into the right striatum (using the bregma as the reference point; 2 mm lateral, 1 mm anterior, and 3 mm deep) to establish an orthotopic GBM model. The mice were engrafted with GBM cell lines as described in the individual experiments. These mice were then sorted before treatment allocation to ensure that the mean Flux values were similar across all treatment groups. OAd or CAR-T cells were intratumorally injected at the indicated time points. Tumor growth was monitored in vivo via bioluminescence imaging using an IVIS Spectrum (PerkinElmer) system after intraperitoneal injection of D-luciferin substrate solution. In some experiments, blood samples were obtained from mice via postorbital venous puncture 10 days after treatment. Cytokine levels were measured using the Mouse Inflammation Array Kit (FAM-INF-1-48, RayBiotech) and the Human IL15 ELISA Kit (EHC013, NeoBioscience) following the manufacturer’s guidelines. The body weight and survival status of the mice were monitored regularly. Bioluminescence signals were quantified using Living Image 4.4 (PerkinElmer).

### Statistical analyses

The differences between or among groups were determined by Student’s t-test or ANOVA. Survival curves were analyzed by conducting the log-rank (Mantel-Cox) test. Statistical significance was set at a *p* < 0.05. The data are presented as the mean ± SD, and all analyses and visualizations were conducted using the GraphPad software (version 9.4).

## Results

### Effective delivery of OAd-GFP via tumor-targeted CAR-T cells

**Fig. 1:**
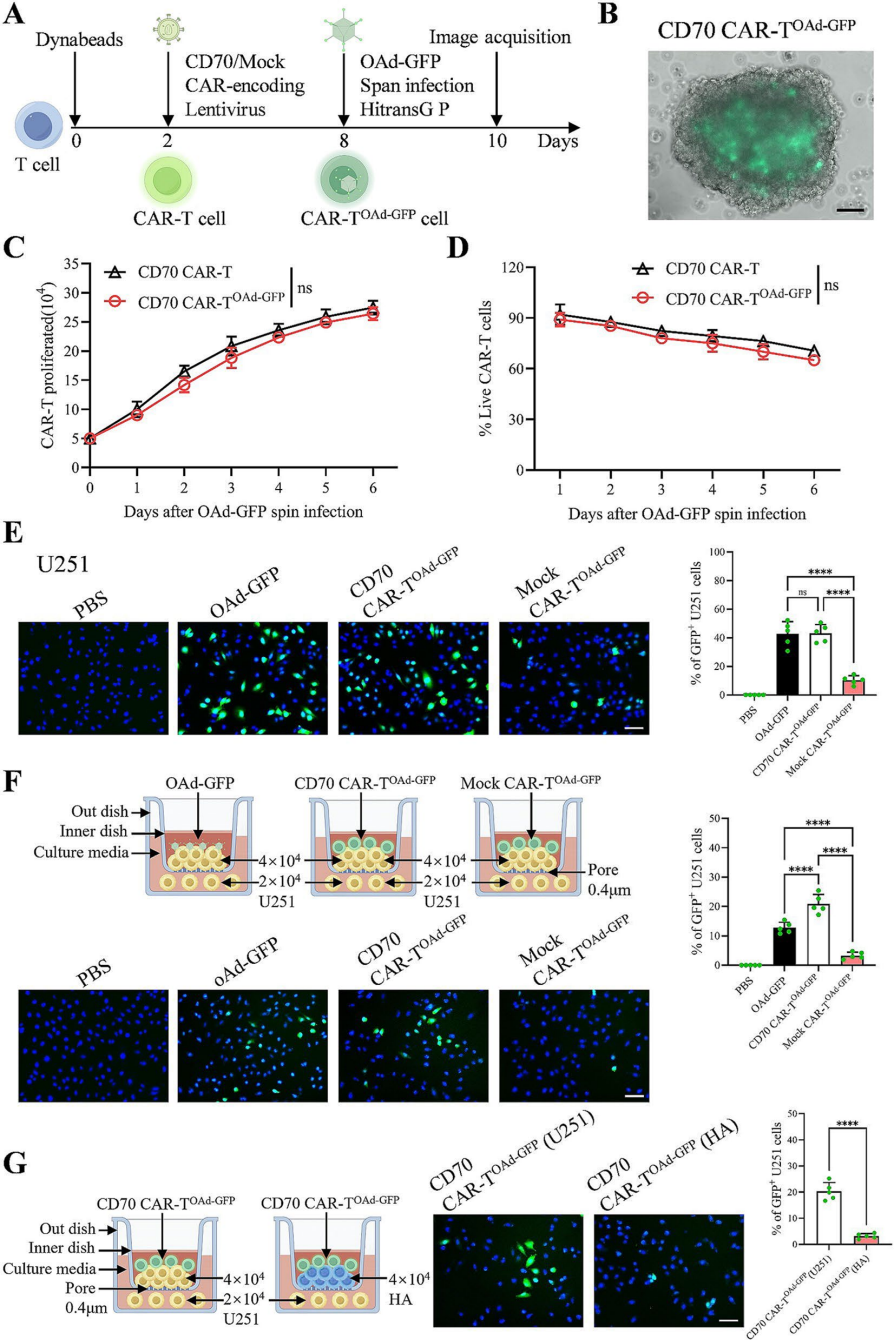
OAd-GFP delivery mediated via CD70 CAR-T cells. (**A**) Procedure for generating CAR-T^OAd−GFP^ cells. OAd was added to CAR-T cells at an MOI of 400:1 with 1× HitransG P, followed by centrifugation at 2100 rpm for 90 min. (**B**) CAR-T cells were observed using a fluorescence microscope 48 h after OAd-GFP was added; scale bar: 50 μm. (**C**) The expansion of CAR-T and CAR-T^OAd−GFP^ cells (*n* = 3 donors/group). (**D**) The percentage of viable CAR-T^OAd−GFP^ cells was quantified via a trypan blue assay (*n* = 3 donors/group). (**E**) OAd-GFP (1.2 × 10^7^VP), CD70 CAR-T^OAd−GFP^ (2 × 10^4^) or Mock CAR-T^OAd−GFP^ (2 × 10^4^) was mixed with U251 cells (4 × 10^4^). Representative images and the proportion of GFP^+^ U251 cells at 24 h are presented; scale bar: 100 μm (*n* = 5/group). (**F**) U251 cells were plated in the top (4 × 10^4^) and bottom (2 × 10^4^) compartments. OAd-GFP (1.2 × 10^7^VP), CD70 CAR-T^OAd−GFP^ (2 × 10^4^), or Mock CAR-T^OAd−GFP^ (2 × 10^4^) cells were added exclusively to the top compartments. Representative images and the proportion of GFP^+^ U251 cells in the bottom compartment were detected after 48 h; scale bar: 100 μm (*n* = 5/group). (G) U251 (2 × 10^4^) cells were plated in the bottom compartments, and CD70 CAR-T^OAd−GFP^ (2 × 10^4^) cells were seeded in the top compartments. HA (4 × 10^4^) or U251 cells (4 × 10^4^) were added to the top compartments. Representative images are shown, and the proportion of GFP^+^ U251 cells in the bottom compartments was detected after 48 h; scale bar, 100 μm (*n* = 5/group). The data are presented as the mean ± SD; ns = no significance and ****p < 0.0001 via unpaired Student’s t-test (**G**), two-way ANOVA with Sidak’s multiple comparisons test (**C** and **D**), or one-way ANOVA with Tukey’s multiple comparisons test (**E** and **F**)

Although OAd-GFP, a genetically engineered third-generation OAd (Fig. S1A), selectively infects and lyses GBM cells [29], whether non-tumor cells, like tumor-targeted CAR-T cells, can be infected by OAd-GFP remains uncertain. Human CD70 or Mock CAR-T cells, with about 55% CAR-positive cells (Figs. 1A, S1B, S1C, and S1D), were generated and cultured for six days. Adenoviruses enter cells via interactions between their fiber proteins and coxsackie-adenovirus receptor (CXADR) on the cell surface [39]. The results of the flow cytometry analysis revealed that CXADR expression was lower in CAR-T cells than in GBM cells and HA (Fig. S1E). However, the results of qPCR assays revealed that Ki67 mRNA was highly expressed in CAR-T cells (Fig. S1F), and the results of Western blotting assays indicated stable expression of the TGF-β2 protein in CAR-T cells, although its expression level was lower in these cells than in GBM cells (Fig. S1G). These results indicated that once OAd-GFP infects CAR-T cells, it can replicate within them.

As shown in Fig. S2A, after eight days of expansion of activated T cells, directly adding OAd-GFP to T cells at multiplicities of infection (MOIs) of 100:1, 200:1, 400:1, or 800:1 without spin infection for 48 h did not lead to significant OAd-GFP infection in T cells. To infect T cells with OAd-GFP, we followed a protocol resembling the lentiviral transduction method used in CAR-T-cell production, utilizing HitransG P (a less toxic alternative to polybrene) and performing centrifugation at 2100 rpm for 90 min. The OAd-GFP infection rate in T cells increased to 30% at an MOI of 400:1 via this alternative method (Fig. S2A). Although the addition of HitransG P and centrifugation for 90 min did not affect T-cell proliferation significantly, OAd-GFP-infected T (T^OAd−GFP^) cells showed reduced proliferation (Fig. S2B) and progressively declining viability (Fig. S2C) about six days after OAd-GFP infection at an MOI of 800:1. In contrast, at an MOI of 400:1, OAd-GFP had a limited effect on T-cell proliferation and viability (Figs. S2B, S2C, and S2D), with the OAd-GFP positivity rate in T cells remaining at 17.8% even seven days post-infection (Fig. S2E). We added OAd-GFP to CD70 CAR-T cells that had expanded for six days at an MOI of 400:1, along with HitransG P, and centrifuged them for 90 min (Fig. 1A). The proliferation and viability of CD70 CAR-T cells infected with OAd-GFP (CAR-T^OAd−GFP^) did not decrease significantly at six days post-infection (Fig. 1B and C, and 1D). These findings showed that there is a seven-day window during which CAR-T^OAd−GFP^ cells can deliver OAd-GFP into GBM cells.

We next examined whether CAR-T^OAd−GFP^ cells could deliver OAd-GFP to GBM cells and whether this process depended on the tumor antigen specificity of CAR-T cells. For CD70^+^ U251 cells (Fig. S3A), CD70 CAR-T^OAd−GFP^ cells, at an effector-to-target (E: T) ratio of 1:2, effectively delivered OAd-GFP into the cells, unlike Mock CAR-T^OAd−GFP^ cells (Fig. 1E). A Transwell assay was conducted to exclude trogocytosis [40] as a potential mechanism for transferring the GFP gene. GFP^+^ U251 cells were observed in the bottom compartment (Fig. 1F), probably because CD70 CAR-T^OAd−GFP^ cells, rather than Mock CAR-T^OAd−GFP^ cells, were reactivated by U251 cells in the top compartment, leading to the release of OAd-GFP and subsequent diffusion to the U251 cells below. However, adding CD70 CAR-T^OAd−GFP^ cells to CD70^-^ HA (Fig. S3A) did not cause infection of U251 cells in the bottom compartment (Fig. 1G). These results highlighted that CAR-T^OAd−GFP^ cells efficiently delivered OAd-GFP to GBM cells in a target antigen-dependent manner.

### OAd-GFP-infected CAR-T cells exhibit superior anti-GBM efficacy

**Fig. 2:**
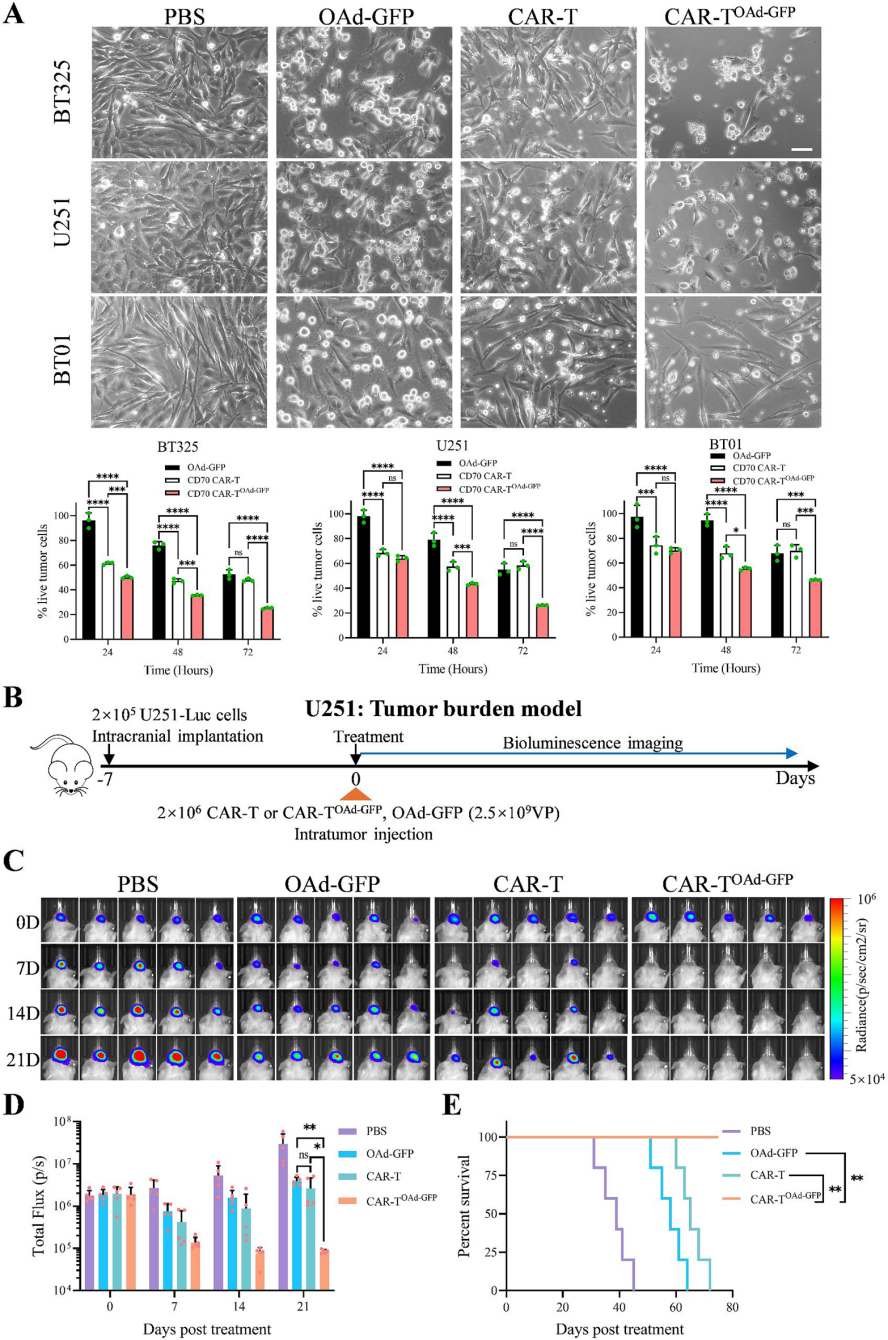
CAR-T^OAd−GFP^ cells show strong antitumor efficacy in vitro and in a tumor burden model of GBM. (**A**) GBM cells (2 × 10^4^) were treated with OAd-GFP (6 × 10^6^VP), CD70 CAR-T cells (2 × 10^4^) or CD70 CAR-T^OAd−GFP^ cells (2 × 10^4^). Cytotoxicity assays were conducted at 24, 48, and 72 h. Representative images at 72 h and percentages of live tumor cells at 24, 48, and 72 h (*n* = 3/group) are shown. (**B**) Treatment schematic: For the GBM tumor burden model, NCG mice (*n* = 5/group) were injected with 2 × 10^5^ U251-Luc cells and treated intratumorally with CAR-T (2 × 10^6^), CAR-T^OAd−GFP^ (2 × 10^6^), or OAd-GFP (2.5 × 10^9^) seven days after injection (day 0). (**C**) Bioluminescence images at the specified time points are presented. (**D**) Flux (p/s) was measured in each experimental group at the specified time points. (**E**) Post‑treatment survival curve. The data are presented as the mean ± SD; ns = no significance, *p < 0.05, **p < 0.01, ***p < 0.001, and ****p < 0.0001 via two-way ANOVA with Tukey’s multiple comparisons test (**A**), one-way ANOVA with Tukey’s multiple comparisons test (**D**), or log-rank (Mantel-Cox) test (**E**)

We subsequently evaluated the antitumor activity of OAd-GFP-infected CAR-T cells by conducting in vitro cytotoxicity assays. An E: T ratio of 1:1 was used for CAR-T cells and GBM cells. First, the cytotoxic activity of CD70 CAR-T cells against various GBM cell lines was evaluated. The expression of CD70 was detected in GBM cells but was not detected in HA (Fig. S3A). The percentage of CD70^+^ cells followed the order BT325 = U251 > BT01, whereas the intensity of CD70 expression was the highest in BT325, followed by that in U251 and BT01 (Figs. S3B and S3C). These differences highlighted the antigenic heterogeneity of GBM cells. After co-culturing CAR-T cells with GBM cells for 24 h, CD70 CAR-T cells showed the strongest cytotoxicity against BT325 cells, while the cytotoxicity against CD70-low-expressing BT01 cells was low (Fig. S3D). The results of a simple linear regression analysis suggested that CD70 CAR-T-cell-mediated killing may depend on the density of CD70 targets and the percentage of CD70^+^ cells. (Figs. S3E and S3F). Increasing the E: T ratio to 4:1 improved the cytotoxicity of CD70 CAR-T cells to target cells (Fig. S3G). After adding CD70 CAR-T^OAd−GFP^ cells to GBM cells, the antitumor efficacy against GBM cells progressively increased over 72 h and significantly surpassed that of CD70 CAR-T cells following 72 h of co-culture (Fig. 2A). In BT01 cells, CD70 CAR-T^OAd−GFP^ significantly enhanced the in vitro antitumor efficacy compared to that of CD70 CAR-T cells (Fig. 2A).

To investigate the ability of CD70 CAR-T^OAd−GFP^ cells to eradicate pre-established tumors in vivo, an NCG mouse tumor burden model was constructed (intracranial orthotopic injection of U251 cells, with control group mice having an approximate survival time of 40 days after the initiation of treatment), followed by intratumoral injection of CD70 CAR-T^OAd−GFP^ (Fig. 2B). Although intratumoral injection of OAd-GFP or CD70 CAR-T cells suppressed tumor progression and prolonged mouse survival, the CAR-T^OAd−GFP^ combination had a strong antitumor effect, leading to tumor regression and long-term survival (Fig. 2C and D, and 2E). These results indicated that CAR-T^OAd−GFP^ cells exhibit robust anti-GBM efficacy and can overcome CAR-T-cell therapy resistance caused by antigen heterogeneity.

### CAR-T cells infected with oad encoding IL15 show improved expansion, persistence, and cytolytic activity in vitro

The cytotoxicity of CD70 CAR-T cells against GBM cells did not significantly increase with prolonged co-culture time (Fig. 2A). We hypothesized that this may be due to changes in the state of CAR-T cells during their co-culture with target cells. Additionally, as OAd-GFP carrier cells, these changes might decrease the cytotoxic effectiveness of CAR-T^OAd−GFP^. The expression levels of exhaustion markers in CD70 CAR-T cells were assessed following their co-culture with CD70^+^ GBM cells. T-cell exhaustion markers were significantly upregulated after 72 h of antigen stimulation, whereas their upregulation remained relatively limited after 24 h of co-culture (Fig. S4A). When co-cultured with CD70– GBM cells (Fig. S4B) instead of with CD70^+^ GBM cells for 72 h, T-cell exhaustion markers were not significantly upregulated (Fig. S4C). When CD70 CAR-T cells were co-cultured with CD70^+^ GBM cells and supplemented with IL2, IL7, IL12, and IL15, we found that IL7 and IL12 did not increase CD70 CAR-T-cell cytotoxicity (Fig. S5A). IL2 promoted the cytolysis of CD70 CAR-T cells, although the difference was not significant (Fig. S5A). In contrast, IL15 enhanced the cytotoxicity of CD70 CAR-T cells (but not Mock CAR-T cells) and decreased the level of expression of T-cell exhaustion markers under antigen stimulation (Figs. S5A, S5B, and S5C). These results indicated that IL15 can enhance the cytotoxic effect of GBM-specific CAR-T cells and alleviate CAR-T-cell exhaustion under continuous antigen stimulation.

**Fig. 3:**
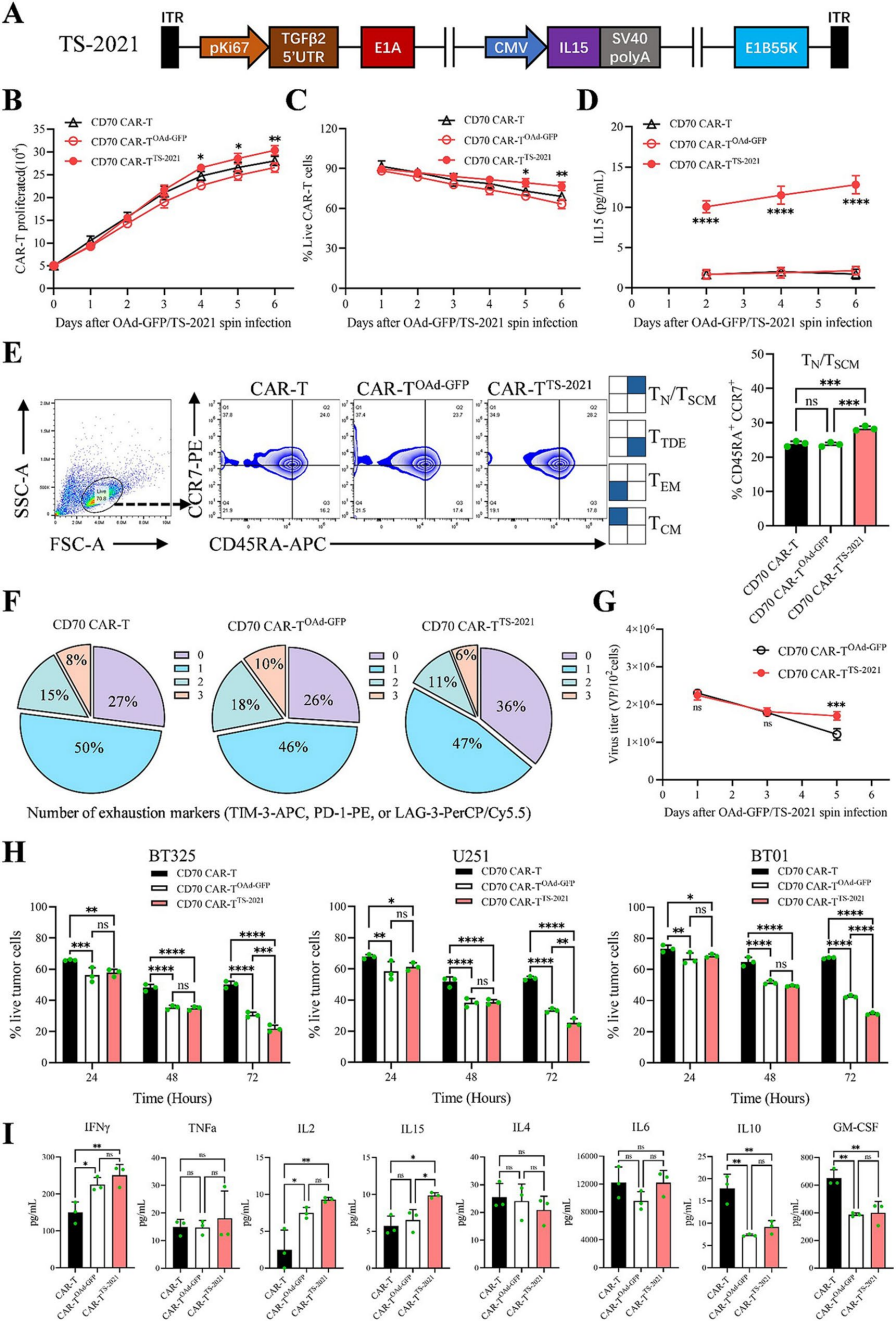
CAR-T^TS−2021^ cells exhibit enhanced proliferation, persistence, and cytolytic capacity in vitro. (**A**) Schematic illustration of the TS-2021 construct, in which E1B55K remains at its native locus. (**B**) The expansion of CAR-T, CAR-T^OAd−GFP^, and CAR-T^TS−2021^ cells (*n* = 3 donors/group). (**C**) The percentages of live CAR-T^OAd−GFP^ and CAR-T^TS−2021^ cells were quantified by the trypan blue assay (*n* = 3 donors/group). (**D**) IL15 secretion was quantified in the supernatants from CAR-T, CAR-T^OAd−GFP^, and CAR-T^TS−2021^ cells by ELISA (*n* = 3 donors/group). (**E**) Flow cytometry plots distinguishing naive/stem cell memory T cells (CD45RA^+^CCR7^+^), central memory T cells (CD45RA^−^CCR7^+^), effector memory T cells (CD45RA^−^CCR7^−^), and terminal differentiation effect memory cells (CD45RA^+^CCR7^–^). The proportion of T cells in the naive/memory stem cell state is presented. CAR-T-cell phenotypic characterization five days after adding OAd-GFP/IL15 (*n* = 3 donors/group). (**F**) Expression of exhaustion markers in CAR-T, CAR-T^OAd−GFP^, and CAR-T^TS−2021^ cells after three days of coincubation with U251 cells. Pie charts illustrate the percentage of cells expressing 0, 1, 2, or 3 exhaustion markers (PD-1, LAG-3, or TIM-3), the average of three different donors. (**G**) CAR-T^OAd−GFP^ and CAR-T^TS−2021^ cells were isolated and lysed to extract viral DNA at the indicated time points, after which OAd-GFP/IL15 titers were determined by qPCR (*n* = 3 donors/group). (**H**) GBM cells (2 × 10^4^) were treated with OAd-GFP (6 × 10^6^VP), CD70 CAR-T cells (2 × 10^4^), CD70 CAR-T^OAd−GFP^ (2 × 10^4^), or CD70 CAR-T^TS−2021^ (2 × 10^4^). Cytotoxicity assays were conducted at 24, 48, and 72 h. The percentages of live tumor cells are shown (*n* = 3/group). (**I**) Multiplex cytokine profiling of supernatants was performed on samples isolated from CAR-T, CAR-T^OAd−GFP^, and CAR-T^TS−2021^ cells stimulated with U251 cells for 48 h (E: T = 1:1, *n* = 3 donors/group). The data are presented as the mean ± SD; ns = no significance, *p < 0.05, **p < 0.01, ***p < 0.001, and ****p < 0.0001 via two-way ANOVA with Tukey’s multiple comparisons test (**B**, **C**, **D**, and **H**), two-way ANOVA with Sidak’s multiple comparisons test (**G**), or one-way ANOVA with Tukey’s multiple comparisons test (**E** and **I**)

TS-2021 is a modified version of OAd-GFP in which IL15 replaces GFP (Fig. 3A). When GBM cells are infected, TS-2021 facilitates the synthesis and release of IL15 by GBM cells (Figs. S6A and S6B). CD70 CAR-T cells infected with TS-2021 (using the same transfection method as OAd-GFP) showed higher proliferative capacity and viability within six days post-infection (Fig. 3B and C). IL15 secretion was detectable in the supernatant starting from day 2 post-infection (Fig. 3D). On day 5 post-infection, flow cytometry was performed to evaluate the differentiation status of various CAR-T cells. Compared to CAR-T cells, CAR-T^TS−2021^ had a greater proportion of naive/stem cell memory T cells (CD45RA^+^CCR7^+^) [41], whereas CAR-T^OAd−GFP^ did not exhibit this characteristic (Fig. 3E). After 72 h of co-culture with CD70^+^ U251 cells, CAR-T^OAd−GFP^ cells presented an exhaustion phenotype similar to that of CAR-T cells, whereas CAR-T^TS−2021^ showed a lower expression of exhaustion markers (Fig. 3F). The OAd load in CAR-T cells was dynamically assessed post-infection via qPCR, and the results showed that the average OAd load in CAR-T^TS−2021^ cells increased after five days (Fig. 3G). When different CD70 CAR-T cells were incubated with GBM cells (E: T = 1:1) for 72 h, CAR-T^TS−2021^ exhibited superior cytolytic activity compared to CAR-T^OAd−GFP^ (Fig. 3H). In vitro cytokine production was assessed in CAR-T, CAR-T^OAd−GFP^, and CAR-T^TS−2021^ cells co-cultured with CD70^+^ U251 cells. In OAd-infected CAR-T cells, IFNγ and IL2 levels increased, whereas IL10 and GM-CSF levels decreased. IL15 levels increased only in TS-2021-infected CAR-T cells. The secretion levels of TNFα, IL4, and IL6 were not significantly different among the three cell types (Fig. 3I). Unlike CAR-T cells, OAd-infected CAR-T cells did not induce excessive cytokine release under in vitro conditions. These findings indicated that CAR-T^TS−2021^ cells showed greater proliferation, persistence, and cytotoxicity in vitro than CAR-T^OAd−GFP^ cells. Additionally, OAd-infected CAR-T cells did not induce significant cytokine release toxicity in vitro.

### CAR-T^TS-2021^ cells exhibit greater antitumor efficacy and lower cytokine release toxicity than CAR-T^OAd-GFP^ cells

**Fig. 4:**
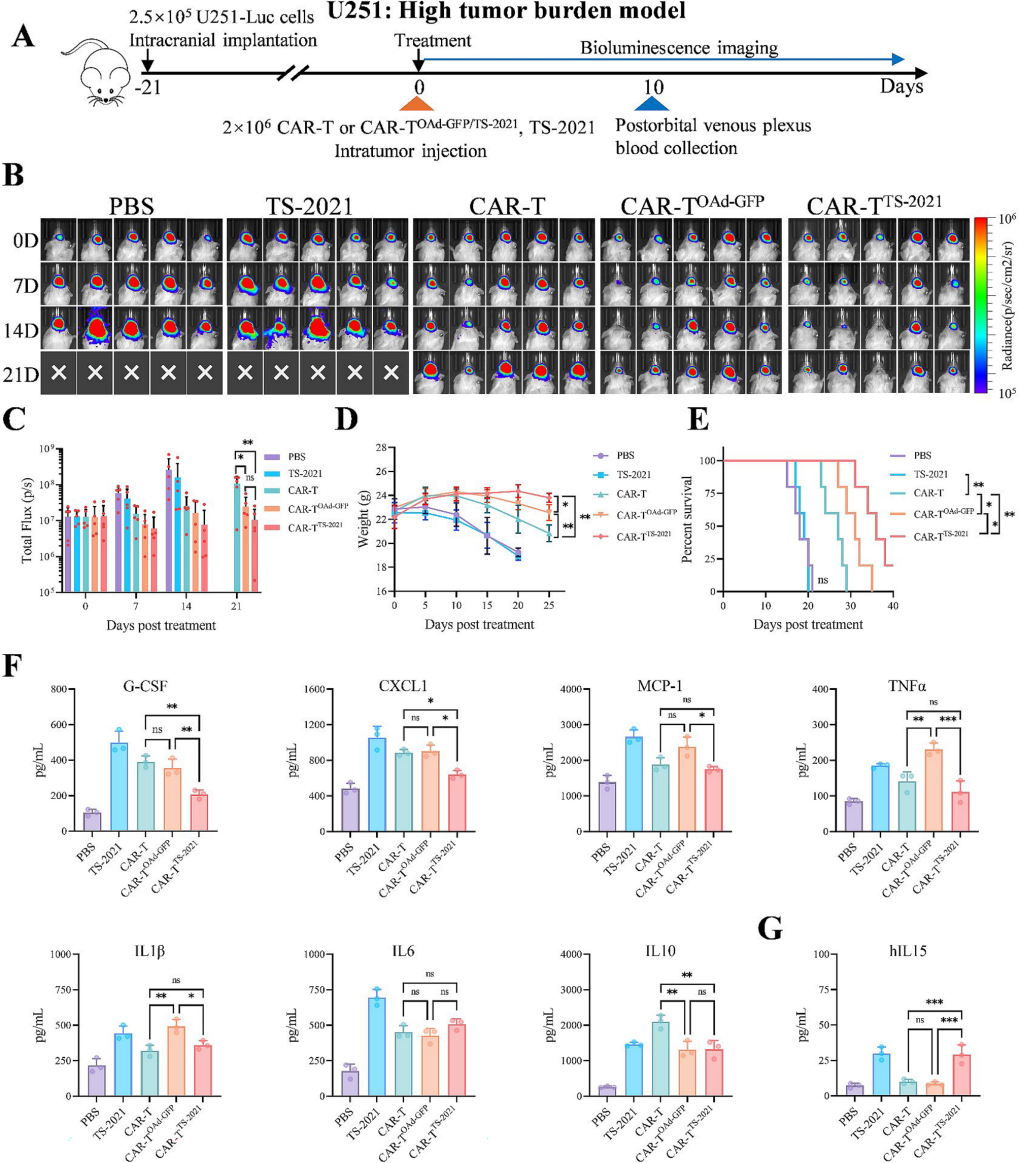
CAR-T^TS−2021^ cells exhibit strong tumor-targeting efficacy and minimal cytokine release toxicity in a high tumor burden model of GBM. (**A**) Treatment schematic: In a high tumor burden model of GBM, NCG mice (*n* = 5/group) were injected with 2.5 × 10^5^ U251-Luc cells and subsequently treated (intratumoral injection) with CAR-T cells (2 × 10^6^), CAR-T^OAd−GFP^ (2 × 10^6^), CAR-T^TS−2021^ (2 × 10^6^), or TS-2021 (2.5 × 10^9^) 21 days post-injection (day 0). (**B**) Bioluminescence images of GBM xenografts at the specified time points are presented. (**C**) Measurement of flux (p/s) in each experimental group at the specified time points. (**D**) The body weights of the mice in the experimental groups were recorded. (**E**) Post-treatment survival curves for each group. (**F**) Evaluation of cytokine levels in the peripheral blood of mice 10 days after treatment (*n* = 3/group). (**G**) Evaluation of hIL15 levels in the peripheral blood of mice 10 days after treatment (*n* = 3/group). The data are presented as the mean ± SD; ns = no significance, *p < 0.05, **p < 0.01, ***p < 0.001, and ****p < 0.0001 via one-way ANOVA with Tukey’s multiple comparisons test (**C**, **D**, **F**, and **G**) or log-rank (Mantel-Cox) test (**E**)

We subsequently evaluated the in vivo antitumor efficacy of CAR-T^TS−2021^ cells. Based on the strong performance of CD70 CAR-T^OAd−GFP^ cells in the NCG mouse tumor burden model, we established a high tumor burden model by intracranially injecting U251 cells into NCG mice (the mice in the control group had a survival time of about 20 days post-treatment). CD70 CAR-T^TS−2021^ cells were then administered via intratumoral injection, and blood samples were obtained via postorbital venous puncture 10 days after treatment to measure cytokine levels (Fig. 4A). In the high-tumor burden model, TS-2021 showed low efficacy (Fig. 4B). CD70 CAR-T-cell therapy delayed tumor growth and extended survival, probably due to the high expression of CD70 in U251 cells. Both CD70 CAR-T^OAd−GFP^ and CD70 CAR-T^TS−2021^ intratumoral injections strongly inhibited tumor growth and preserved mouse body weight compared to CD70 CAR-T-cell injections. Compared to that in the CAR-T^OAd−GFP^ group, the survival time of the mice in the CAR-T^TS−2021^ group was significantly longer (Fig. 4C and D, and 4E). In contrast to the intratumoral injection of CD70 CAR-T cells into mice, CD70 CAR-T^TS−2021^ treatment did not increase serum cytokine levels. Mice treated with CD70 CAR-T^TS−2021^ cells showed reduced cytokine levels in the serum compared to those treated with CAR-T^OAd−GFP^ cells (Fig. 4F). Additionally, human IL15 (hIL15), which is essential for sustaining CAR-T-cell activity, was identified in the peripheral blood of mice in the CD70 CAR-T^TS−2021^ group (Fig. 4G). These results indicated that CAR-T^TS−2021^ cells exhibited stronger in vivo anti-GBM efficacy than CAR-T^OAd−GFP^ cells, while CAR-T cells infected with TS-2021 did not induce increased cytokine release toxicity.

### IL15 mitigates the DNA damage induced by oad in CAR-T cells

**Fig. 5:**
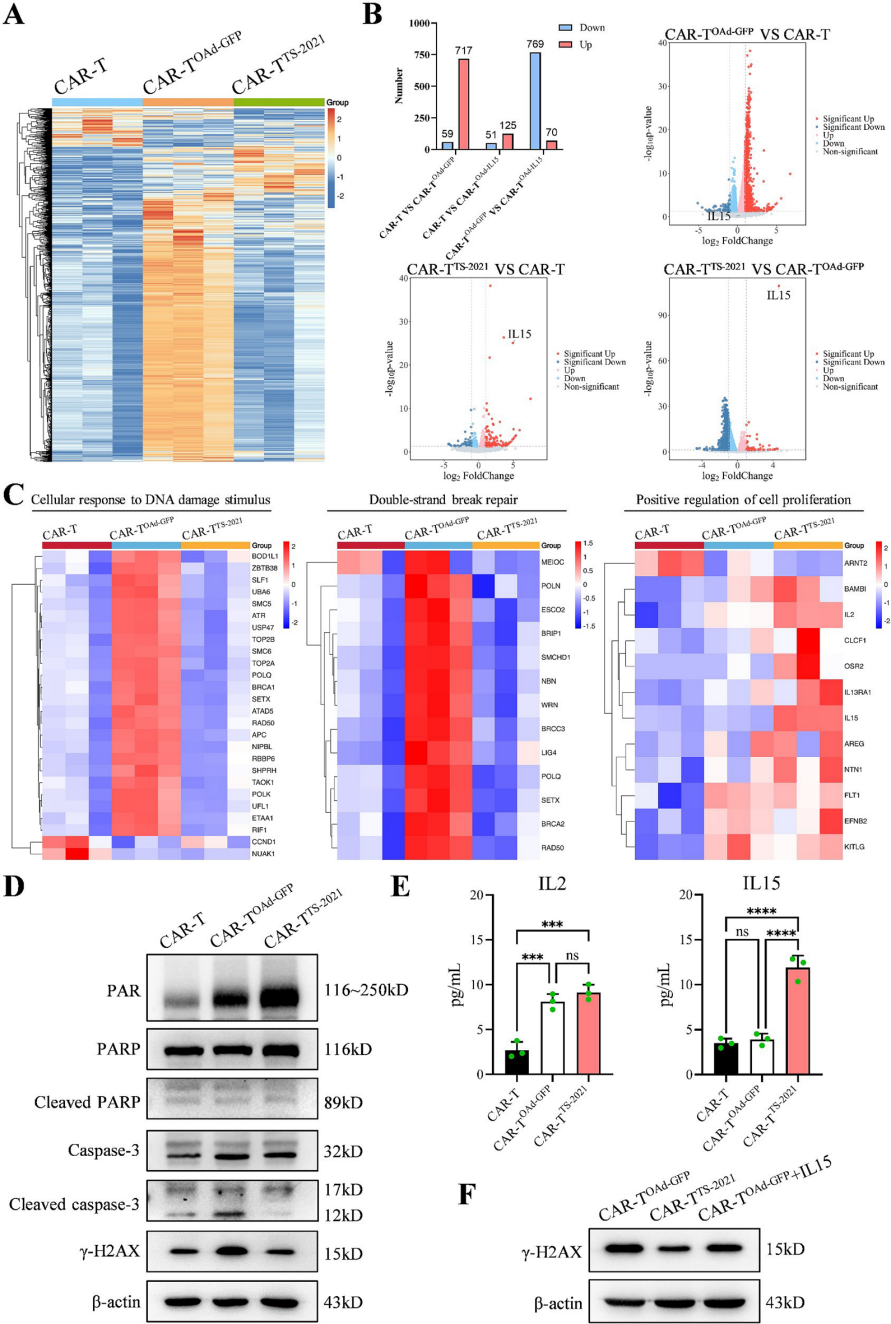
CAR-T^TS−2021^ protects T cells from OAd-induced DNA damage through IL15 expression. (**A**) Hierarchical clustering of gene expression in CAR-T, CAR-T^OAd−GFP^, and CAR-T^TS−2021^ cells three days after adding OAd-GFP/IL15 (*n* = 3 donors/group). (**B**) Volcano plots show the quantification and p-value of differentially expressed genes (*n* = 3 donors/group). (**C**) Genes enriched in three terms (cellular response to DNA damage stimulus, double-strand break repair, and positive regulation of cell proliferation) according to the GO enrichment analysis. (**D**) CAR-T, CAR-T^OAd−GFP^, and CAR-T^TS−2021^ cell lysates were assessed by Western blotting. (E) IL2 and IL15 concentrations in CAR-T, CAR-T^OAd−GFP^, and CAR-T^TS−2021^ cells (*n* = 3 donors/group) were determined by ELISA. (**F**) CAR-T^OAd−GFP^ cells were stimulated with IL15 for three days, followed by Western blotting analysis. The data are presented as the mean ± SD; ns = no significance, ***p < 0.001, and ****p < 0.0001 via one-way ANOVA with Tukey’s multiple comparisons test (**E**)

To better understand how TS-2021 infection enhances the antitumor efficacy of CAR-T cells, we conducted RNA sequencing on CD70 CAR-T, CD70 CAR-T^OAd−GFP^, and CD70 CAR-T^TS−2021^ cells. A clustering heat map was made to illustrate the differences in gene expression among various CAR-T cells (Fig. 5A). The differential gene volcano plot revealed that OAd-GFP, but not TS-2021, had a stronger effect on the changes in gene expression in CAR-T cells (Fig. 5B). GO analysis was performed on the genes with differential expression, highlighting the top 16 most significantly enriched GO terms. In CAR-T^TS−2021^ cells, the term “positive regulation of cell proliferation” was prominent, whereas in CAR-T^OAd−GFP^ cells, the terms “cellular response to DNA damage stimulus” and “double-strand break repair” were prominent (Fig. S7). DNA damage-related genes were primarily upregulated in CAR-T^OAd−GFP^, whereas IL15 was upregulated only in CAR-T^TS−2021^. IL2 was expressed in both CAR-T^OAd−GFP^ and CAR-T^TS−2021^ (Fig. 5C). The results of the immunoblotting analysis revealed that γ-H2AX, a DNA damage marker for double-strand breaks [42], was highly expressed in CAR-T^OAd−GFP^ cells, whereas cleaved caspase-3, a marker of apoptosis [43], was expressed at the lowest level in CAR-T^TS−2021^ cells (Fig. 5D). The expression of poly-ADP-ribose (PAR) in CAR-T^OAd−GFP^ and CAR-T^TS−2021^ cells suggested that OAd entry into CAR-T cells activated the poly (ADP-ribose) polymerase (PARP) repair function. Additionally, the higher expression of PARP in CAR-T^TS−2021^ cells indicated increased repair efficiency, which was consistent with the increased expression of PAR in these cells. PARP expression was downregulated in CAR-T and CAR-T^OAd−GFP^ cells because of cleavage by cleaved caspase-3 (Fig. 5D). Consistent with the RNA sequencing results, IL15 synthesis increased only in CAR-T^TS−2021^ cells, whereas IL2 expression was upregulated in CAR-T^OAd−GFP^ and CAR-T^TS−2021^ cells, as determined by cytokine assays of the supernatant (Fig. 5E). The level of expression of γ-H2AX in CAR-T^OAd−GFP^ cells was lower after 72 h of IL15 supplementation than in cells without IL15 treatment (Fig. 5F). These results indicated that OAd infection induces DNA damage in CAR-T cells, whereas IL15 effectively alleviates this damage.

### IL15 sustains the expansion and viability of CAR-T^TS-2021^ cells through the JAK-STAT and MAPK-ERK signaling pathways

**Fig. 6:**
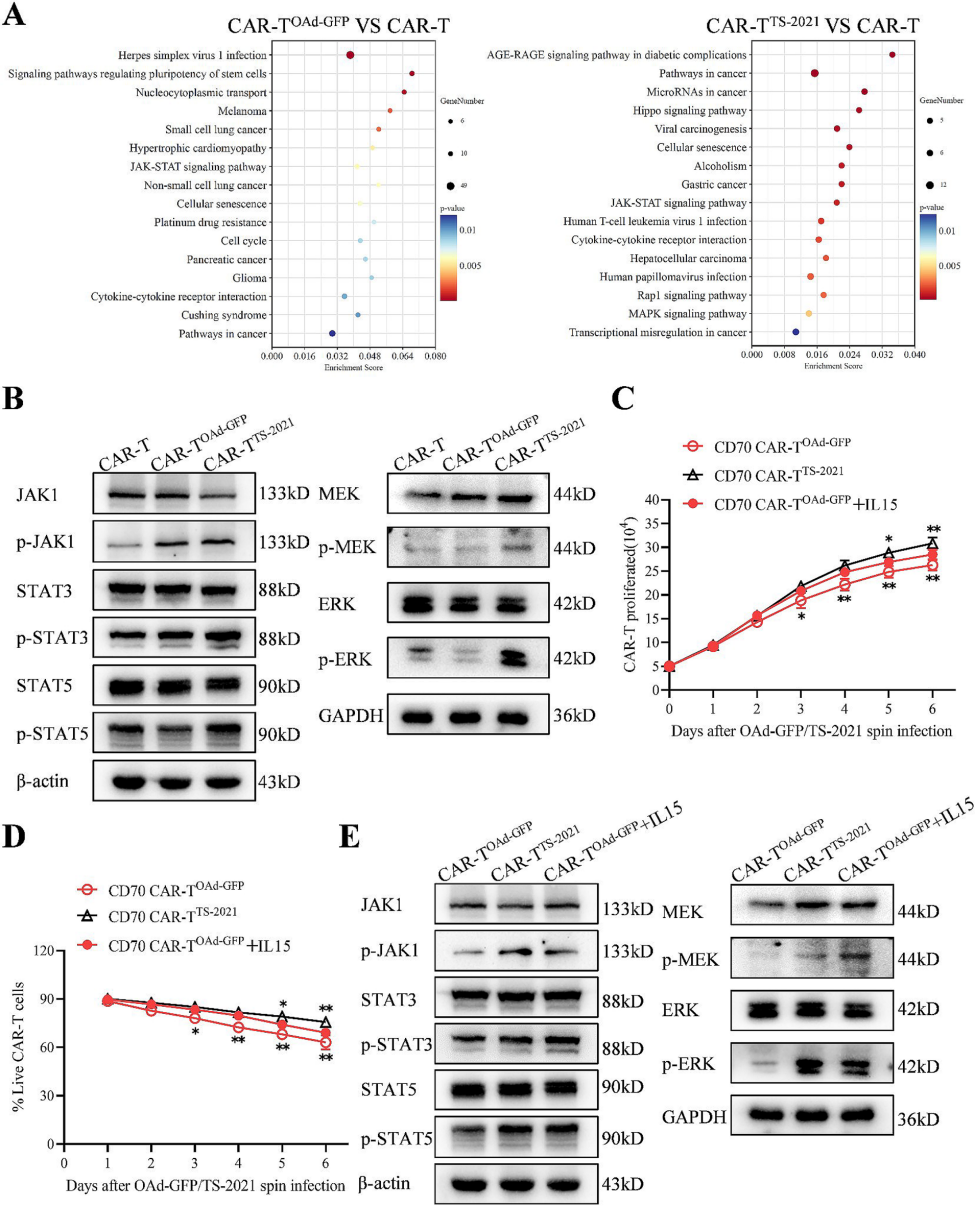
CAR-T^TS−2021^ cells maintain proliferation and viability through activation of the JAK-STAT and MAPK signaling pathways. (**A**) Enrichment analysis of KEGG pathways associated with genes differentially expressed between CAR-T cells and CAR-T^OAd−GFP/IL15^ cells. The top 16 pathways are shown according to the p-value (*n* = 3 donors/group). (**B**) Western blotting analysis of JAK1, STAT3, STAT5, MEK, and ERK expression in CAR-T, CAR-T^OAd−GFP^, and CAR-T^TS−2021^ cells. (**C**) The expansion of CAR-T^OAd−GFP^ after treatment with IL15 (*n* = 3 donors/group). (**D**) Percentages of live CAR-T^OAd−GFP^ after treatment with IL15 were quantified by the trypan blue assay (*n* = 3 donors/group). (**E**) Western blotting analysis of JAK1, STAT3, STAT5, MEK, and ERK expression in CAR-T^OAd−GFP^ cells after treatment with IL15. The data are presented as the mean ± SD; *p < 0.05 and **p < 0.01 via two-way ANOVA with Tukey’s multiple comparisons test (**C** and **D**)

To further assess how IL15 mitigates OAd-induced DNA damage in CAR-T cells, we conducted the Kyoto Encyclopedia of Genes and Genomes (KEGG) enrichment analysis of differentially expressed genes and highlighted the 16 most significantly enriched pathways (Fig. 6A). Pathways involved in T-cell proliferation have received significant attention. In this study, the JAK-STAT and MAPK pathways were enriched in CAR-T^TS−2021^ cells. In contrast, CAR-T^OAd−GFP^ was enriched primarily in pathways associated with viral infection. The JAK-STAT pathway was also enriched, probably due to the synthesis and release of IL2 induced by viral infection. Next, we confirmed the results of the differential gene KEGG enrichment analysis by conducting Western blotting assays (Fig. 6B). Both p-JAK1 and p-STAT3 were upregulated in CAR-T^OAd−GFP^ and CAR-T^TS−2021^ cells, suggesting that the JAK-STAT pathway was activated in CAR-T cells following OAd infection. In contrast, p-STAT5, p-MEK, and p-ERK were significantly expressed only in CAR-T^TS−2021^, probably due to the synthesis and release of IL15. IL15 was added to the culture medium of CAR-T^OAd−GFP^ cells. The results revealed that CAR-T^OAd−GFP^ enhanced proliferation and viability over six days post-infection, although it lagged behind that of CAR-T^TS−2021^ cells (Fig. 6C and D). The advantage in CAR-T^TS−2021^ cells may be attributed to the continuous release of IL15 in these cells. In the presence of IL15, the levels of p-STAT5, p-MEK, and p-ERK increased in CAR-T^OAd−GFP^ cells (Fig. 6E). These results suggested that IL15 promotes the proliferation and viability of CAR-T^TS−2021^ cells by activating the JAK-STAT and MAPK-ERK signaling pathways, thus reversing OAd-induced DNA damage in CAR-T cells.

### CAR-T^TS-2021^ cells alleviate acquired resistance in GBM

**Fig. 7:**
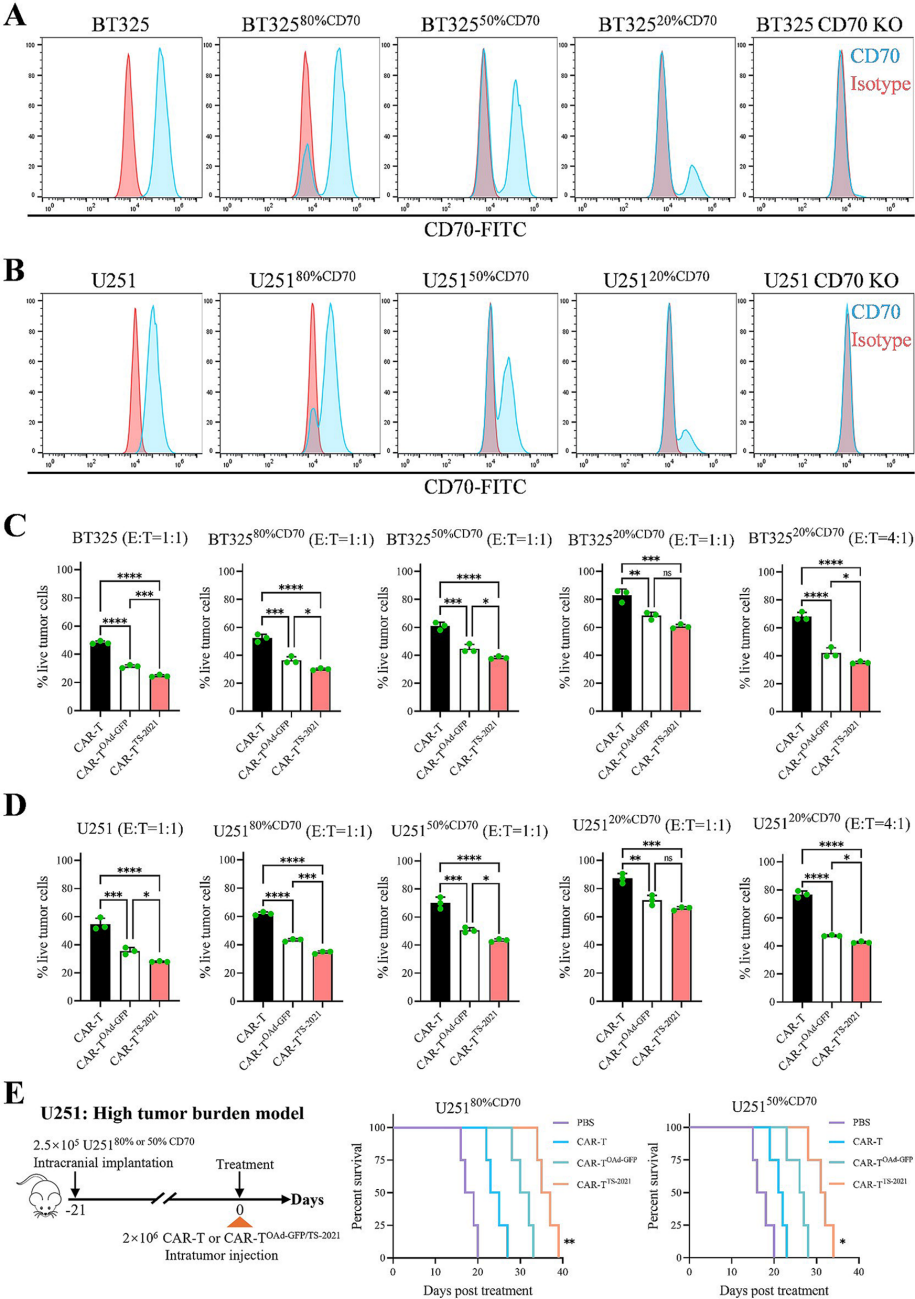
CAR-T^TS−2021^ cells inhibit the growth of GBM cells with antigen heterogeneity both in vivo and in vitro. (**A**) BT325 and BT325-CD70-KO#1 cells were co-cultured at different ratios, after which CD70 expression was detected by flow cytometry. (**B**) FCM assay for CD70 expression in U251 cells after co-culture with U251-CD70-KO#1 cells at different ratios. (**C**) BT325 cells with different CD70 positivity rates were treated with CAR-T, CAR-T^OAd−GFP^, or CAR-T^TS−2021^ cells. Cytotoxicity assays were performed at 72 h. The percentages of live tumor cells are shown (72 h, *n* = 3/group). (**D**) Cytotoxicity assays were performed 72 h after U251 cells with different CD70 positivity rates were treated with CAR-T, CAR-T^OAd−GFP^, or CAR-T^TS−2021^ cells. Percentages of live tumor cells (72 h, *n* = 3/group). (**E**) Treatment schematic: NCG mice (*n* = 4/group) were injected with 2.5 × 10^5^ U251^80%CD70^ or U251^50%CD70^ cells. After tumor growth and randomization, intratumoral treatment with CAR-T cells (2 × 10^6^), CAR-T^OAd−GFP^ (2 × 10^6^), or CAR-T^TS−2021^ (2 × 10^6^) was administered on day 21 post-injection (day 0). Post-treatment survival curve comparing CAR-T^TS−2021^ with other treatment groups. The data are presented as the mean ± SD; ns = no significance, *p < 0.05, **p < 0.01, ***p < 0.001, and ****p < 0.0001 via one-way ANOVA with Tukey’s multiple comparisons test (C and D) or log-rank (Mantel-Cox) test (**E**)

GBM is highly heterogeneous, and mutations or loss of its target antigens can greatly limit the efficacy of CAR-T-cell therapy [44]. GBM cell models were constructed by mixing CD70^+^ and CD70– GBM cells in different proportions (80%, 50%, and 20% CD70^+^) in vitro (Fig. 7A and B) to mimic antigen mutations or loss (AML) in GBM. As the CD70 positivity rate in GBM cells decreased, the cytolytic capacity of CAR-T cells also decreased. At 50% CD70 positivity, OAd-infected CAR-T cells maintained strong antitumor activity, with CAR-T^TS−2021^ demonstrating greater efficacy over CAR-T^OAd−GFP^. However, when the CD70 positivity rate decreased to 20%, the cell-killing ability of all CAR-T cells decreased considerably (Fig. 7C and D). When the E: T ratio increased to 4:1, OAd-infected CAR-T cells exhibited significantly stronger antitumor activity, primarily due to the OAd present in the cells. We intracranially implanted 80% CD70^+^ and 50% CD70^+^ U251 cells to construct a high tumor burden model. NCG mice treated with CAR-T^TS−2021^ survived significantly longer than those in the CAR-T and CAR-T^OAd−GFP^ groups. However, when targeting tumors with 50% antigen positivity, the in vivo tumor-targeting effectiveness of CAR-T^TS−2021^ decreased. These results suggested that CAR-T^TS−2021^ can effectively inhibit GBM growth in AML in vitro and in vivo, partially overcoming AML-associated acquired resistance in GBM.

## Discussion

We developed an innovative strategy to integrate genetically engineered OAd into CAR-T cells. OAd can specifically replicate in CAR-T cells and be selectively delivered to GBM through CAR-T-cell targeting. However, repeated exposure to tumor cell antigens resulted in functional exhaustion of CAR-T cells. The CAR-T^TS−2021^ cells sustained T-cell expansion and persistence by synthesizing IL15 via the exogenous gene carried by TS-2021. This strategy minimized OAd-induced DNA damage in CAR-T cells, increased their anti-GBM efficacy, and helped overcome the therapeutic resistance of GBM.

OAd can selectively replicate in malignant glioma cells through dual regulation by Ki67 and TGF-β2 while theoretically exhibiting minimal or no replication in normal cells [29]. CAR-T cells are highly proliferative and exhibit high Ki67 levels but low TGF-β2 levels. These findings indicate that OAd can replicate in CAR-T cells but with limited efficiency. CAR-T cells infected with OAd avoid rapid lysis, enabling them to survive and maintain their functionality. In this study, directly adding OAd-GFP in vitro did not efficiently infect T cells, probably due to the limited expression of CXADR on the surface of immune cell membranes [45]. Therefore, we followed a lentivirus transduction protocol similar to that used in CAR-T-cell preparation, which included adding the less toxic HitransG P instead of polybrene and centrifuging for 90 min. This process successfully transduced OAd-GFP into T cells. Increasing the MOI of OAd did not significantly increase the transduction efficiency, indicating that OAd overload may compromise the viability of T-cells. To address this, we selected an optimal MOI that balanced high transduction efficiency with minimal damage to T cells, allowing the generation of CAR-T^OAd−GFP^ cells. Although OAd-induced DNA damage was detected in CAR-T^OAd−GFP^ cells, about 70% of CAR-T cells remained uninfected 48 h after exposure to OAd. The results suggested that OAd-GFP-infected CAR-T cells synergized with uninfected CAR-T cells to enhance therapeutic efficacy against GBM. Repeated stimulation by tumor antigens induces functional exhaustion in CAR-T cells [46, 47]; this phenomenon is also observed during co-culture with GBM cells. Cytokines can alleviate T-cell exhaustion [48–50], and IL15 was selected in this study. TS-2021, a modified version of OAd-GFP [29], was transduced into CAR-T cells. CAR-T^TS−2021^ cells showed higher proliferative capacity, viability, and proportion of stem-like cells but lower expression of exhaustion markers under prolonged target antigen stimulation. Together, these attributes contributed to the persistence of CAR-T^TS−2021^ cells. Their virus-carrying capacity also increased substantially. A comprehensive evaluation of T-cell functionality in a preclinical regional administration model requires an “in vivo CAR-T-cell stress test” [51]. To establish an in vivo CAR-T-cell stress test, we constructed a high tumor burden orthotopic model. CAR-T^TS−2021^ cells showed higher anti-GBM efficacy; even when target antigens in GBM were mutated or lost, CAR-T^TS−2021^ cells could still target and eliminate these tumor cells via TS-2021, thus limiting GBM antigen escape.

IL15 primarily regulates downstream effector molecules through the JAK-STAT, PI3K-AKT, and MAPK signaling pathways [52]. It promotes the activation of effector T cells to enhance their antitumor and anti-infection capabilities but also supports the survival and proliferation of memory T cells [53, 54]. However, IL15 does not mediate IL2-induced activation-induced cell death; instead, it inhibits this process [55]. OAd-GFP infection of CAR-T cells induced DNA damage and triggered the PARP repair mechanism. A similar effect has been reported in GBM cells infected with TS-2021 [56]. However, in TS-2021-infected CAR-T cells, autocrine secretion of IL15 increased the proliferation and survival of cells by activating the JAK-STAT and MAPK-ERK pathways, leading to a reduction in CAR-T-cell apoptosis. PARP is cleaved by active caspase-3, resulting in the loss of its ability to mediate DNA damage repair [57, 58]. A decrease in the levels of cleaved PARP may suggest the activation of a more robust PARP repair mechanism in CAR-T^TS−2021^ cells. Compared to the addition of exogenous IL15, continuous intracellular release of IL15 may be a better choice for CAR-T cells. In anti-GBM therapy, CAR-T^TS−2021^ cells can use the IL15 synthesized and released by OAd-infected tumor cells to maintain their functional activity. While this innovative dual-delivery system was specifically designed to improve CAR-T cell persistence through cytokine support, the current study did not incorporate longitudinal in vivo tracking to directly validate this mechanistic hypothesis. We explicitly acknowledge this methodological limitation and propose systematic evaluation of CAR-T survival kinetics as a critical focus for subsequent preclinical investigations.

In this study, CAR-T cells are analogous to a missile, consisting of key components such as a warhead, a guidance system, and a propulsion system. We integrated TS-2021 into CAR-T cells to coordinate and enhance their functions. TS-2021 can use the CAR-T-cell “guidance system” to precisely target tumors, while CAR-T cells increase the “warhead” killing capacity via TS-2021. The IL15 secreted by CAR-T cells is essential for maintaining the “propulsion system” of CAR-T cells. Although combination therapies involving CAR-T cells and oncolytic adenoviruses have been extensively studied and shown promise in solid tumors [59, 60], this integrated delivery strategy may offer potential advantages over conventional approaches that rely on the separate administration of these agents. By embedding viral delivery within the CAR-T cell platform, the CAR-T^TS−2021^ system enables coordinated tumor targeting, synchronized effector activity, and localized payload release within the tumor microenvironment. This design enhances tumor-specific cytotoxicity, optimizes the spatial and temporal co-localization of therapeutic agents, and supports sustained CAR-T cell function through localized IL15 signaling. In central nervous system (CNS) malignancies, preclinical studies have demonstrated that locoregional CAR-T delivery—via intratumoral or intraventricular injection—achieves greater therapeutic efficacy than systemic (intravenous) administration, largely due to the restrictive nature of the blood–brain barrier and the immunosuppressive tumor microenvironment [51, 61]. Informed by these insights, we employed intratumoral administration of CAR-T^TS−2021^ in this study to better address the unique therapeutic challenges posed by CNS tumors. While our in vitro data demonstrated superior anti-GBM activity of CAR-T^TS−2021^ compared to co-administered CAR-T cells and TS-2021 (Fig. S9B), we acknowledge that the absence of a direct in vivo comparison limits the strength of our conclusions. Addressing this limitation will be a priority in future preclinical studies to comprehensively evaluate the therapeutic potential of the CAR-T^TS−2021^ platform.\.

## Conclusions

To summarize, we found that genetically engineered OAd can specifically replicate within CAR-T cells and be precisely transported to GBM via CAR-T cell targeting. TS-2021-infected CAR-T cells used the exogenous gene carried by OAd to facilitate autocrine IL15 secretion, thereby supporting the sustained expansion and persistence of CAR-T cells. This approach demonstrated that CAR-T cells “armed” with TS-2021 may effectively overcome the therapeutic resistance of GBM.

## Electronic supplementary material

Below is the link to the electronic supplementary material.

Supplementary Material 1

Supplementary Material 2

Supplementary Material 3

## Data Availability

No datasets were generated or analysed during the current study.

## Abbreviations

CAR: Chimeric antigen receptor
GBM: Glioblastoma
OAd: Oncolytic adenoviruses
AML: Antigen mutations or loss
TGF-β2: Transforming growth factor-β2
TNF: Tumor necrosis factor
AM: Astrocyte Medium
PBMCs: Peripheral blood mononuclear cells
qPCR: Quantitative polymerase chain reaction
CXADR: Coxsackie-adenovirus receptor
MOIs: Multiplicities of infection
PAR: Poly-ADP-ribose
PARP: Poly (ADP-ribose) polymerase
