# Supplementary material for: CD70 CAR-T cells empowered by TS-2021 through ex vivo transduction show potent antitumor efficacy against glioblastoma
Source: J Exp Clin Cancer Res. 2025 Jun 5;44:173. doi: 10.1186/s13046-025-03431-6 (PMC12139114; doi:10.1186/s13046-025-03431-6)
Supplement: Supplementary file 2 — Supplementary Material 2 [file 13046_2025_3431_MOESM2_ESM.pdf]

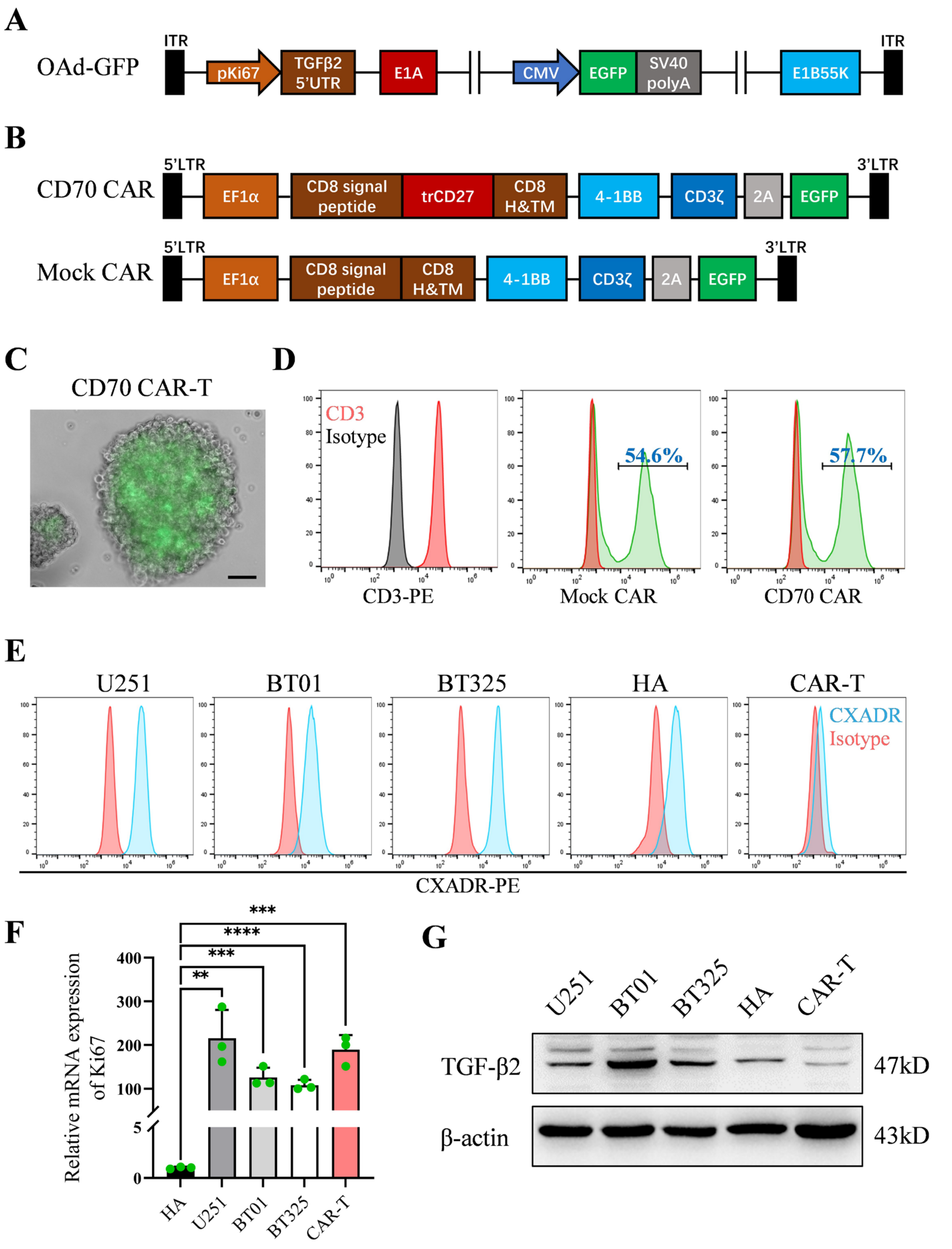

Fig. S1 Theoretical basis for loading OAd-GFP into CAR-T cells.

(A) Schematic illustration of the OAd-GFP construct. (B) A diagram of the CD70 CAR and Mock CAR constructs. (C) Human T cells were observed via fluorescence microscopy six days after transduction with a human CD70 CAR-encoding lentivirus;

scale bar: 50 μm. (D) FCM analysis of CAR expression in CAR-T cells. Data representative of one of five independent experiments are presented. (E) CXADR expression levels were analyzed in GBM cells, HA, and CD70 CAR-T cells by FCM. (F) Relative mRNA expression of Ki67 in GBM cells, HA, and CD70 CAR-T cells was determined by qPCR (n = 3/group). (G) TGF-β2 expression in GBM cells, HA, and CD70 CAR-T cells was measured by Western blotting. The data are presented as the mean ± SD; $^{**}p < 0.01$, $^{***}p < 0.001$, and $^{****}p < 0.0001$ via unpaired Student's t-test (F).

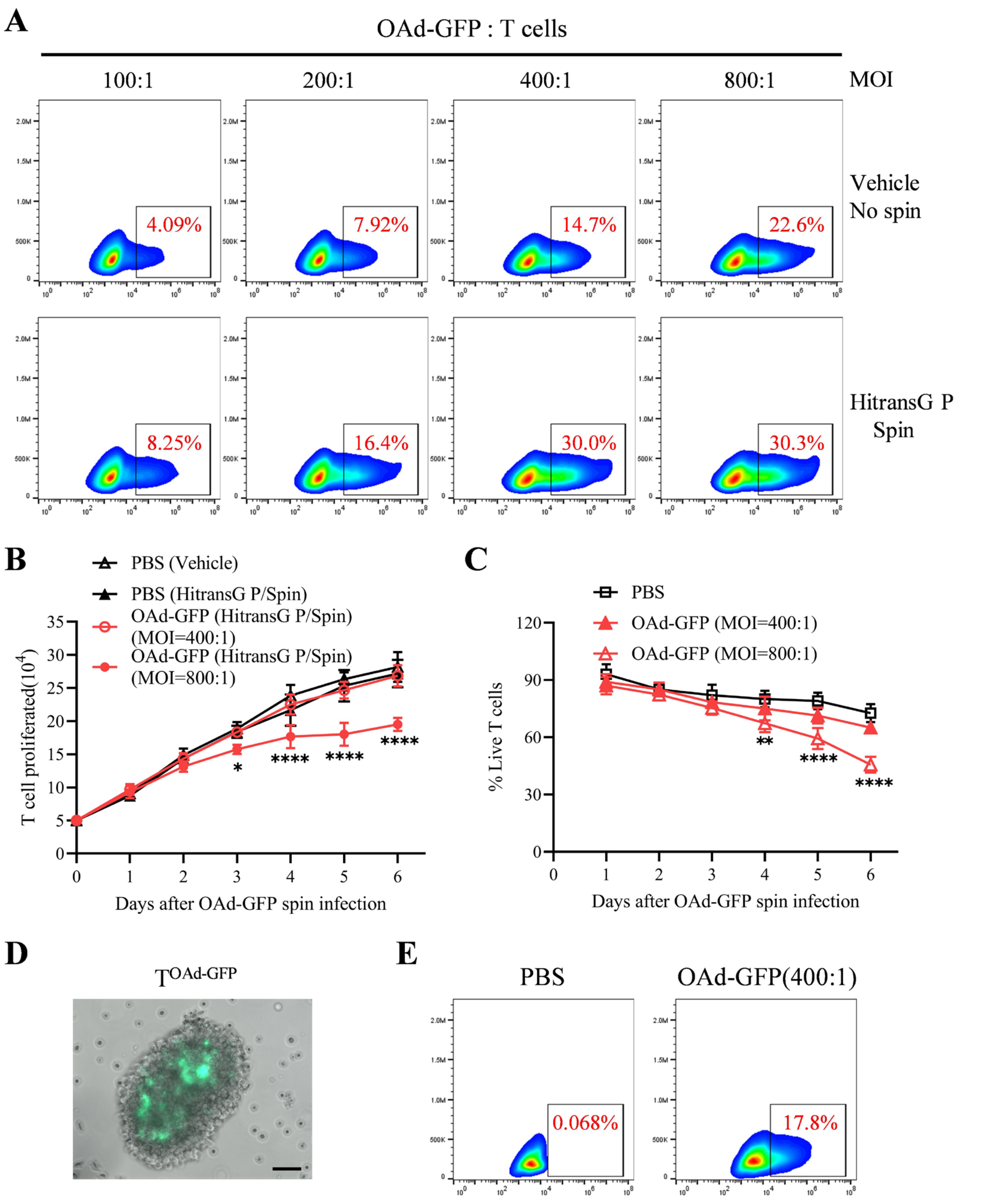

Fig. S2 Optimal conditions for loading OAd-GFP into T cells.

(A) FCM analysis was performed to detect the infection rate 48 h after OAd-GFP was added. Data representative of one of three independent experiments are presented. (B) The expansion of T cells and $T^{OAd\text{-}GFP}$ cells (n = 3 donors/group). (C) Viable $T^{OAd\text{-}GFP}$ cell percentages were quantified by the trypan blue assay (n = 3 donors/group). (D) T cells were observed using a fluorescence microscope 48 h after adding OAd-GFP; scale

bar: 50 μm. (E) OAd-GFP was added to T cells at an MOI of 400:1 with 1× HitransGP, followed by centrifugation at 2100 rpm for 90 min to load OAd-GFP into T cells. The OAd-GFP infection rate was detected by FCM seven days after adding OAd-GFP. Data representative of one of three independent experiments are shown. The data are presented as the mean ± SD; *$p < 0.05$, **$p < 0.01$, and ****$p < 0.0001$ via two-way ANOVA with Tukey's multiple comparisons test (B and C).

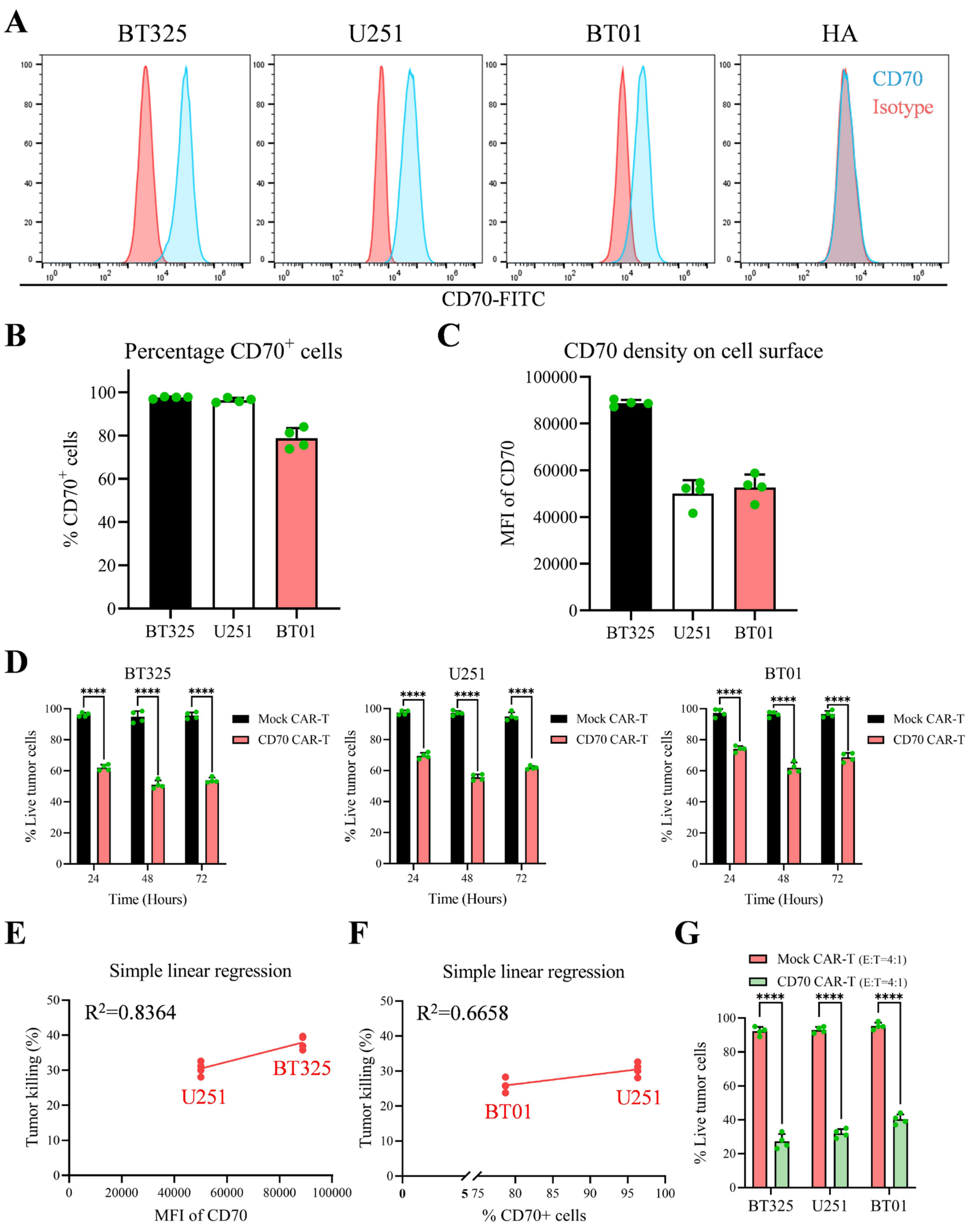

Fig. S3 CD70 CAR-T cells show enhanced antigen-specific killing activity in CD70^{high}-expressing GBM cells.

(A) Representative histograms of CD70 levels in HA and GBM cell lines, as determined by FCM. The data represent one of four independent experiments. (B) Measurement of the proportion of CD70^{+} cells in the BT325, U251, and BT01 cell lines (n = 4/group).

(C) Quantification of CD70 expression intensity (mean fluorescence intensity, MFI) in GBM cell lines (n = 4/group). (D) Percentage of live tumor cells after 24, 48, and 72 h of co-culture with CD70 CAR-T or Mock CAR-T cells (E:T = 1:1, n = 4/group). (E) Evaluation of the relationship between CD70 CAR-T-cell-mediated cytotoxicity (24 h) and the CD70 expression density (MFI) on target cells using simple linear regression. (F) The correlation between CD70 CAR-T-cell-mediated cytotoxicity (24 h) and the percentage of $CD70^+$ cells among target cells was assessed by simple linear regression. (G) Percentages of surviving tumor cells after 24 h of treatment with CD70 CAR-T or Mock CAR-T cells (E:T = 4:1, n = 4/group). The data are presented as the mean ± SD; ****$p < 0.0001$ via unpaired Student's t-test (D and G).

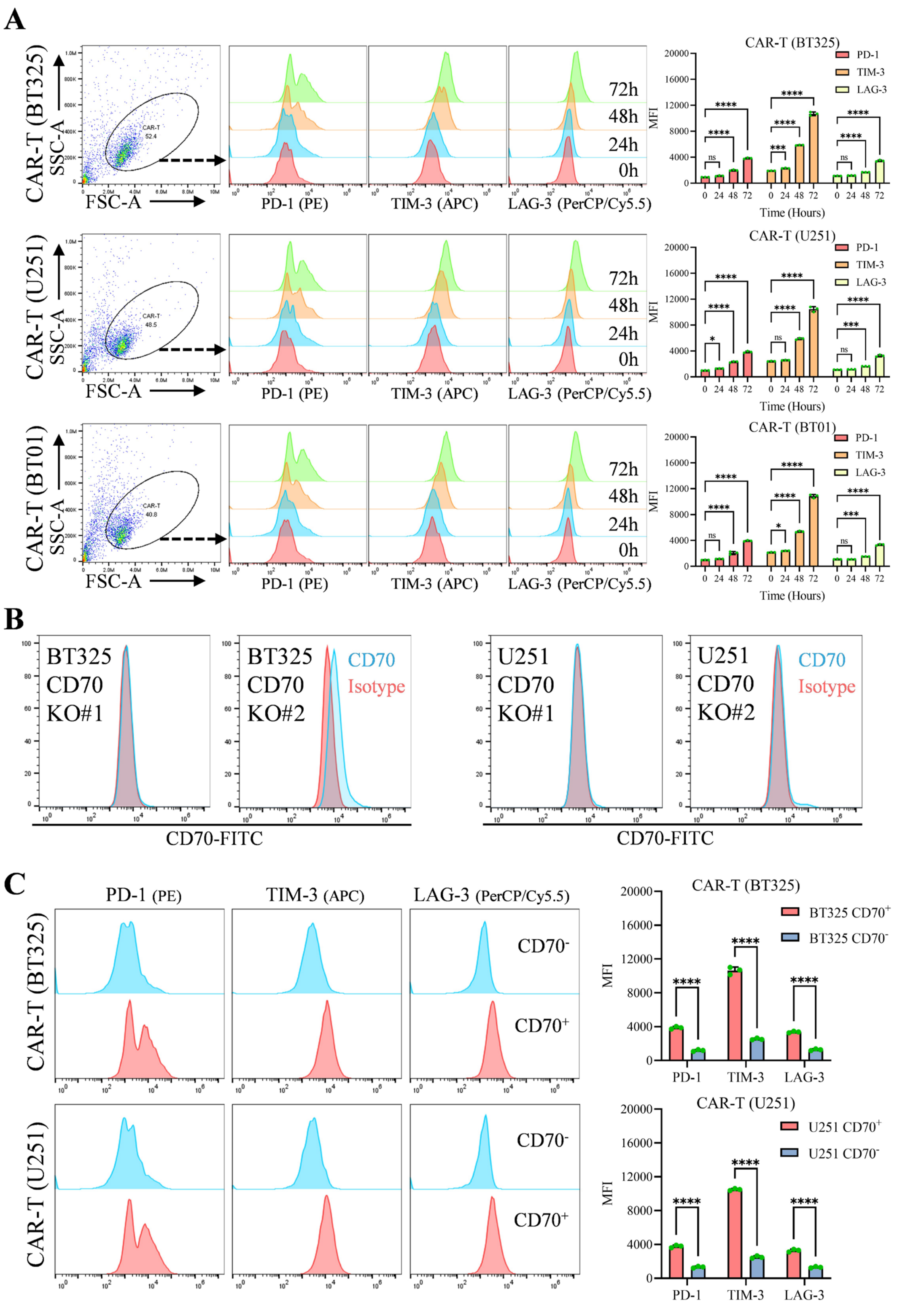

Fig. S4 Continuous antigen stimulation leads to CAR-T-cell exhaustion.

(A) Quantification of PD-1, LAG-3, and TIM-3 expression intensity (MFI) in CD70 CAR-T cells co-cultured with GBM cells at the indicated time points (n = 3/group). (B)

CD70 expression in BT325 and U251 cells was knocked out via CRISPR/Cas9. BT325-CD70-KO#1 and U251-CD70-KO#1 cells were used in subsequent experiments. (C) Quantification of PD-1, LAG-3, and TIM-3 expression intensity (MFI) in CD70 CAR-T cells three days after stimulation with $CD70^{+}$ or $CD70^{-}$ target cells (n = 3/group). The data are presented as the mean ± SD; ns = no significance, *$p < 0.05$, ***$p < 0.001$, and ****$p < 0.0001$ via one-way ANOVA with Tukey's multiple comparisons test (A) or unpaired Student's t-test (C).

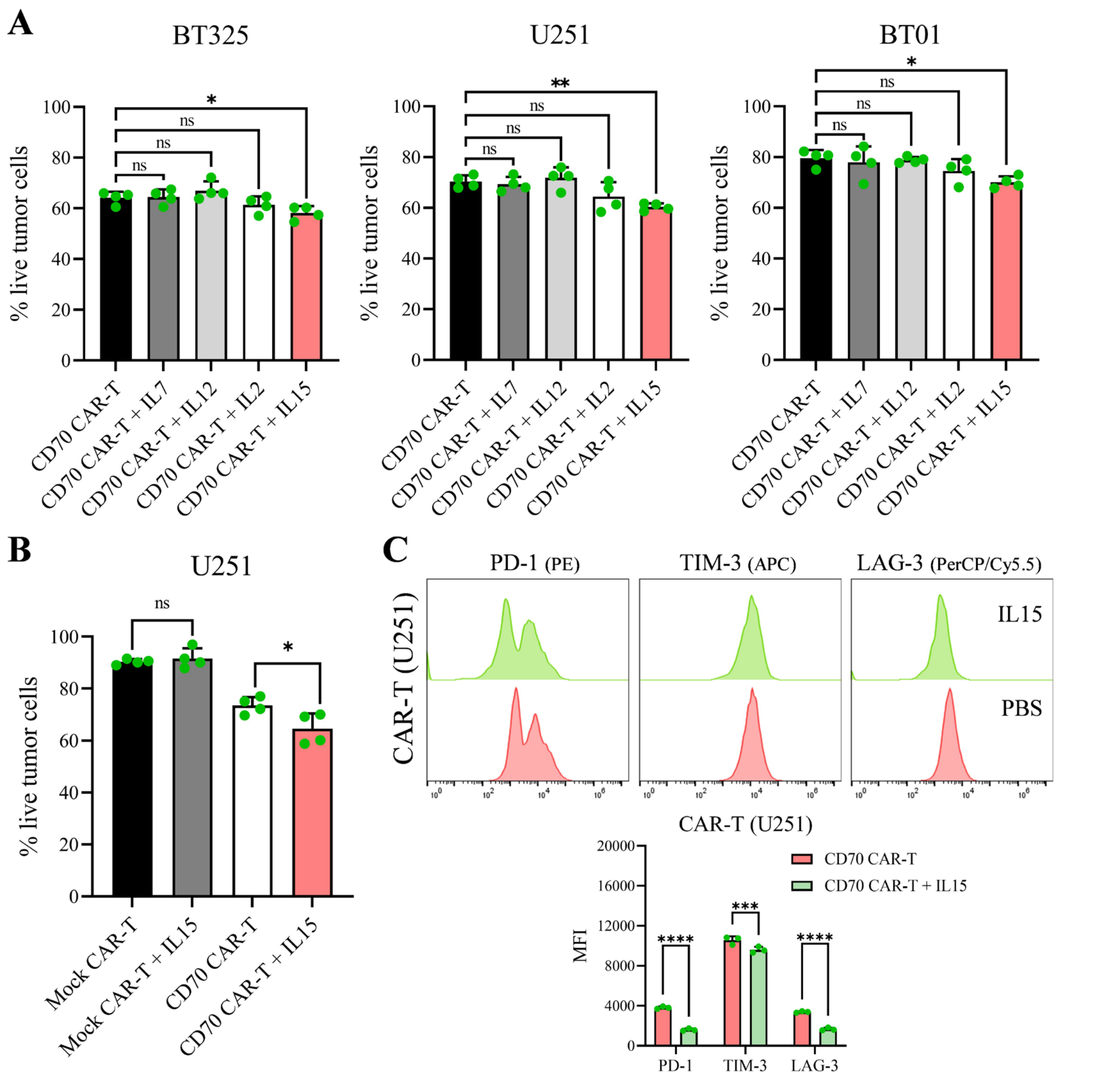

Fig. S5 IL15 enhances the cytotoxicity of CAR-T cells and alleviates their exhaustion under antigen stimulation.

(A) Live tumor cell percentage after 24 h of co-culture with CD70 CAR-T cells or with IL2-, IL7-, IL12-, or IL15-supplemented CD70 CAR-T cells (n = 4/group). (B) Percentages of live U251 cells after 24 h of co-culture with Mock/CD70 CAR-T cells or IL15-stimulated Mock/CD70 CAR-T cells (n = 4/group). (C) Quantification of PD-1, LAG-3, and TIM-3 expression intensity (MFI) in unstimulated or IL15-stimulated CD70 CAR-T cells co-cultured with U251 cells for 72 h (n = 3/group). The data are presented as the mean ± SD; ns = no significance, *$p < 0.05$, **$p < 0.01$, ***$p < 0.001$,

and ****$p < 0.0001$ via unpaired Student's t-test (A, B, and C).

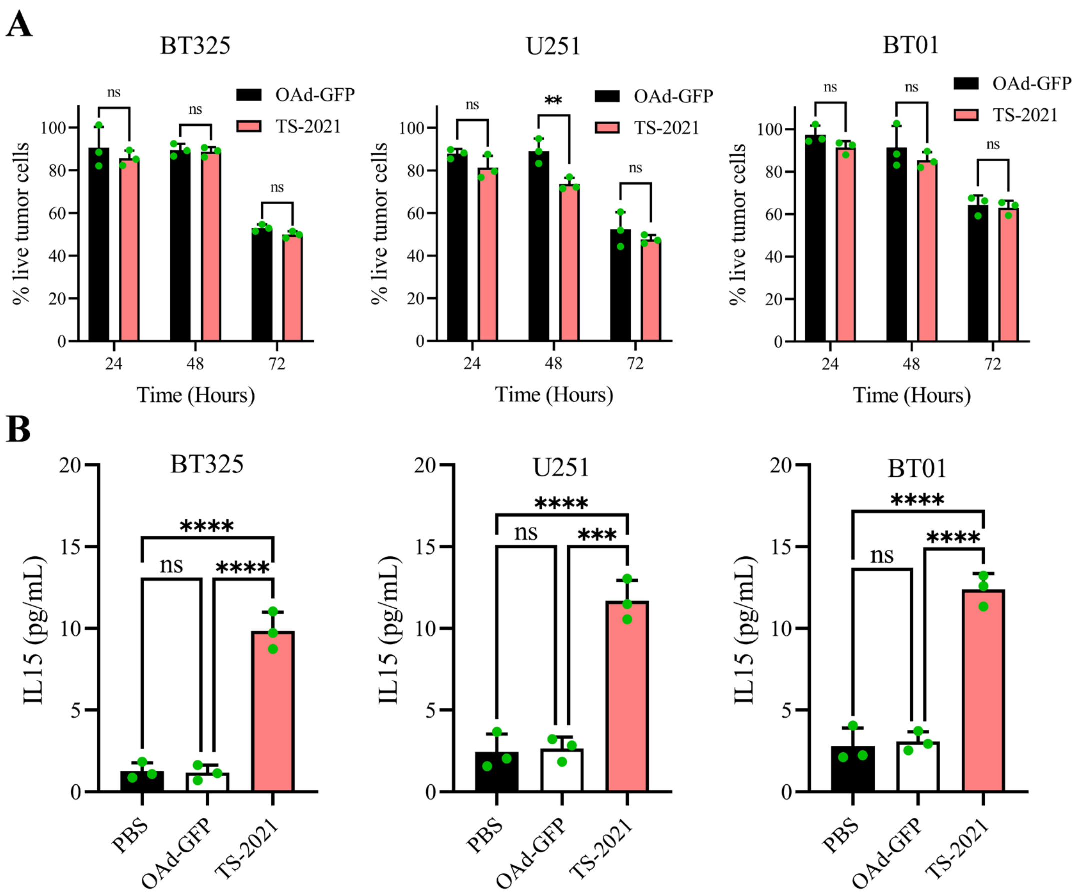

Fig. S6 In vitro antitumor efficacy of OAd-GFP/IL15.

(A) GBM cells were treated with OAd-GFP or TS-2021 (MOI = 300:1). Cytotoxicity assays were conducted at 24, 48, and 72 h. The percentages of live tumor cells are shown (n = 3/group). (B) IL15 secretion was quantified in supernatants from GBM cells co-cultured with OAd-GFP/IL15 (MOI = 300:1) for 72 h by ELISA (n = 3/group). The data are presented as the mean ± SD; ns = no significance, **$p < 0.01$, ***$p < 0.001$, and ****$p < 0.0001$ via two-way ANOVA with Sidak's multiple comparisons test (A) or one-way ANOVA with Tukey's multiple comparisons test (B).

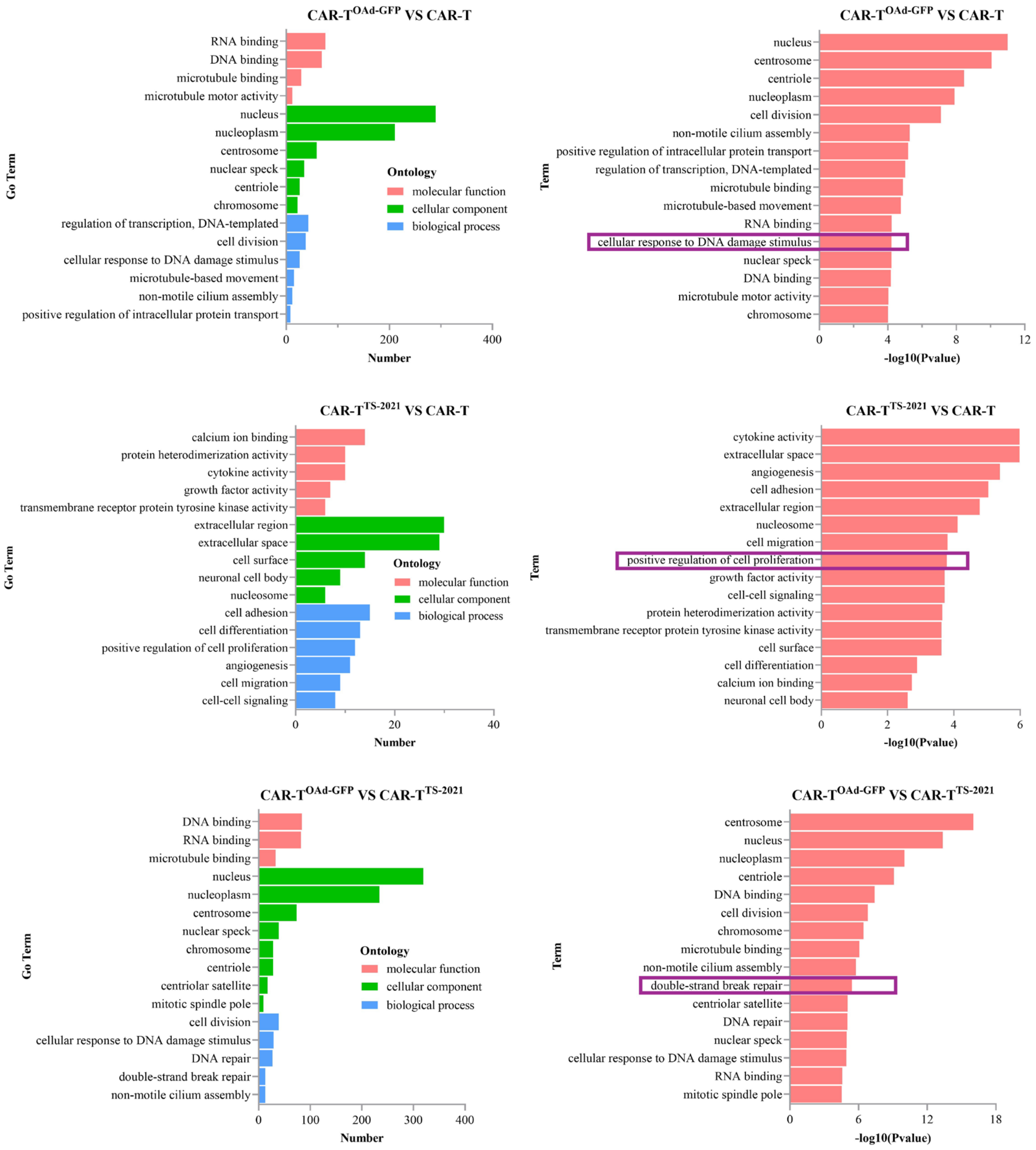

Fig. S7 GO enrichment analysis of RNA sequencing data from CAR-T, CAR-T$^{OAd-GFP}$, and CAR-T$^{TS-2021}$ cells.

GO annotation of differentially expressed genes, showing the top 16 significantly enriched genes across biological processes, cellular components, and molecular functions.

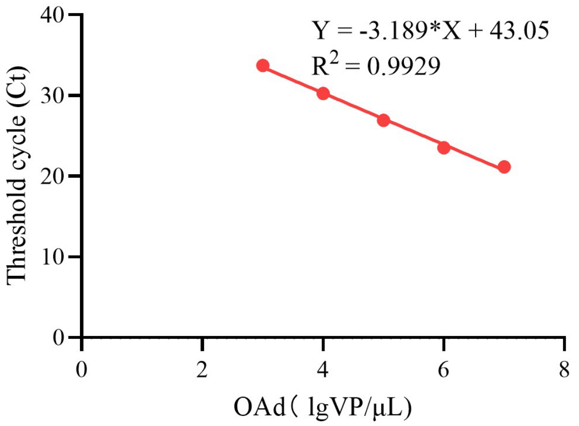

Fig. S8 Quantitative PCR analysis was conducted to determine adenovirus titer.

A standard curve was obtained by plotting the E1A threshold cycle (Ct) values against the initial viral particle (VP) counts of the standards. The linear equation obtained was used to calculate the initial viral titer in unknown samples from their corresponding Ct values.

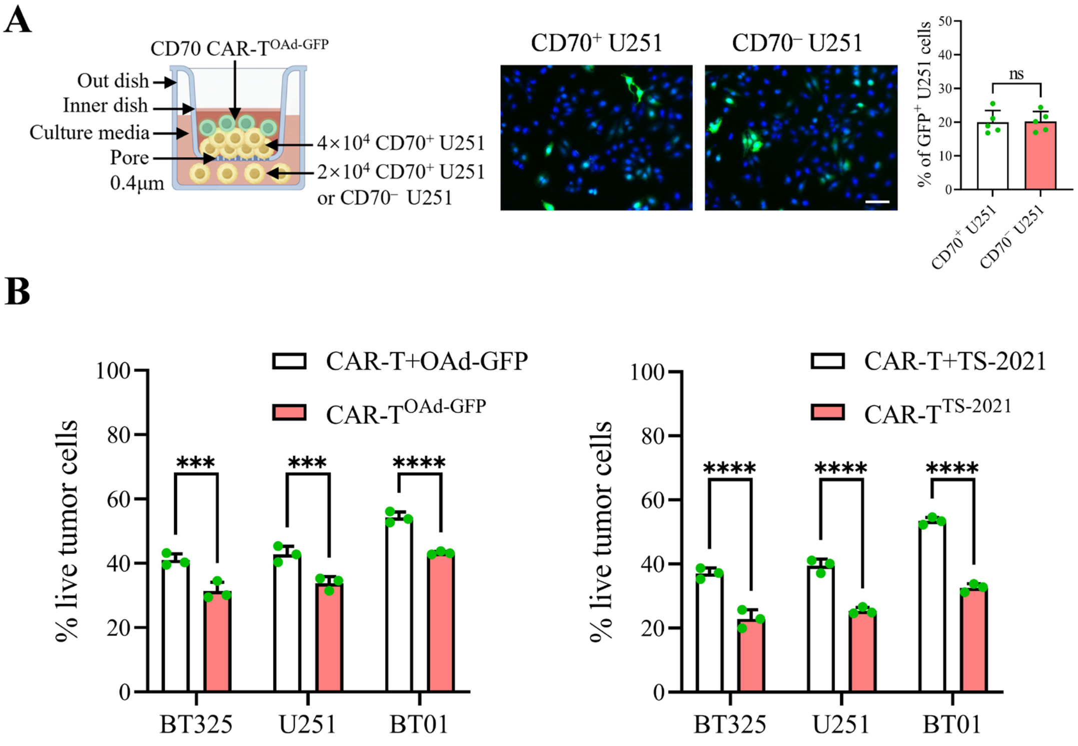

Fig. S9 In vitro evaluation of the antitumor efficacy of CD70 CAR-T^{OAd} compared to the combination of CD70 CAR-T and OAd.

(A) CD70^{+} U251 (2×10^4) or CD70^{-} U251 cells (2×10^4) were plated in the bottom compartments, and CD70 CAR-T$^{OAd\text{-}GFP}$ (2×10^4) cells were seeded in the top compartments. U251 cells (4×10^4) were added to the top compartments. Representative images are shown, and the proportion of GFP^{+} U251 cells in the bottom compartments was detected after 48 h; scale bar, 100 μm (n = 5/group). (B) GBM cells were treated with either CD70 CAR-T^{OAd} or a combination of CD70 CAR-T and OAd at an MOI of 300:1. Cytotoxicity assays were conducted at 72 h. The percentages of live tumor cells are shown (n = 3/group). The data are presented as the mean ± SD; ns = no significance, ***$p < 0.001$, and ****$p < 0.0001$ via unpaired Student's t-test (A and B).
