# Supplementary figures and images for: CD70 CAR-T cells empowered by TS-2021 through ex vivo transduction show potent antitumor efficacy against glioblastoma
Source: J Exp Clin Cancer Res. 2025 Jun 5;44:173. doi: 10.1186/s13046-025-03431-6 (PMC12139114; doi:10.1186/s13046-025-03431-6)

Figure S1G

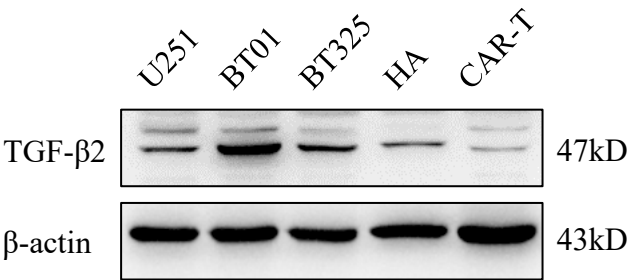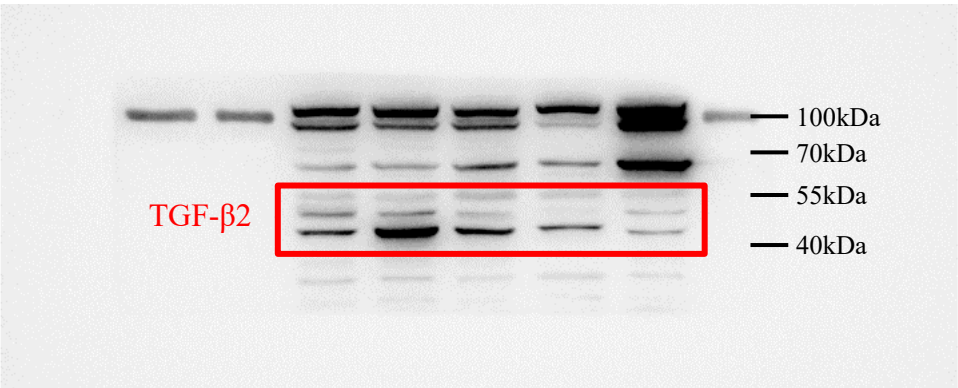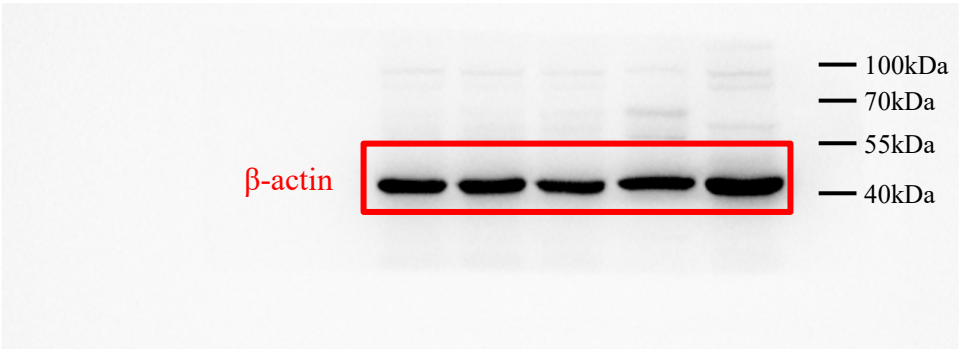

Figure 5D

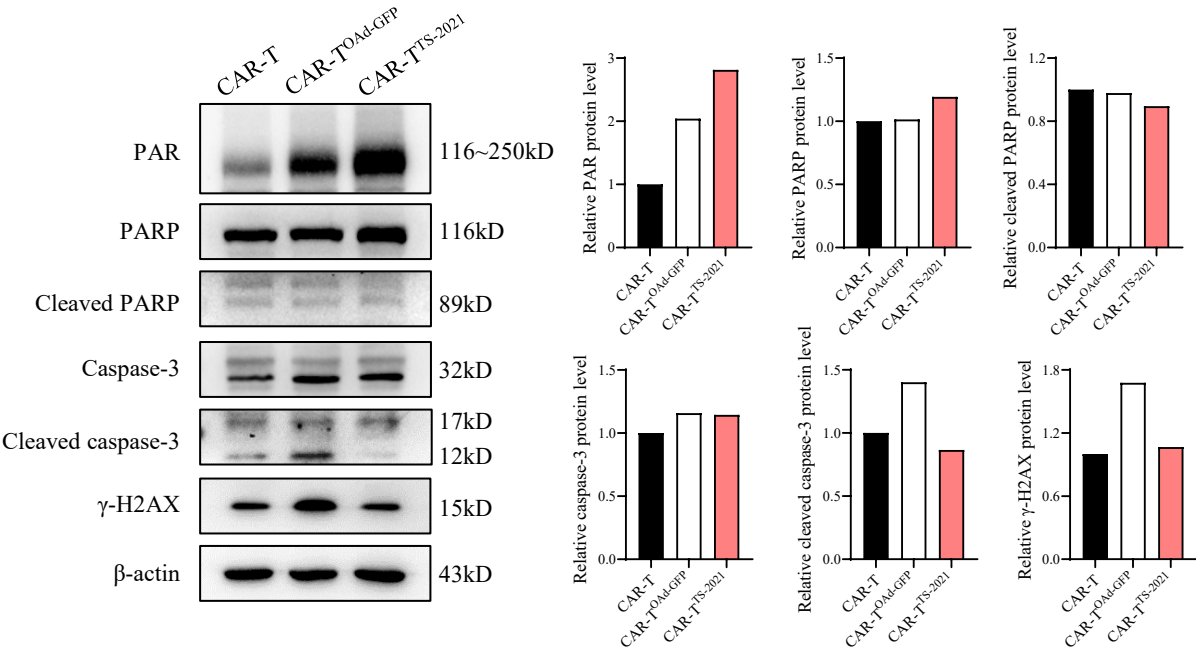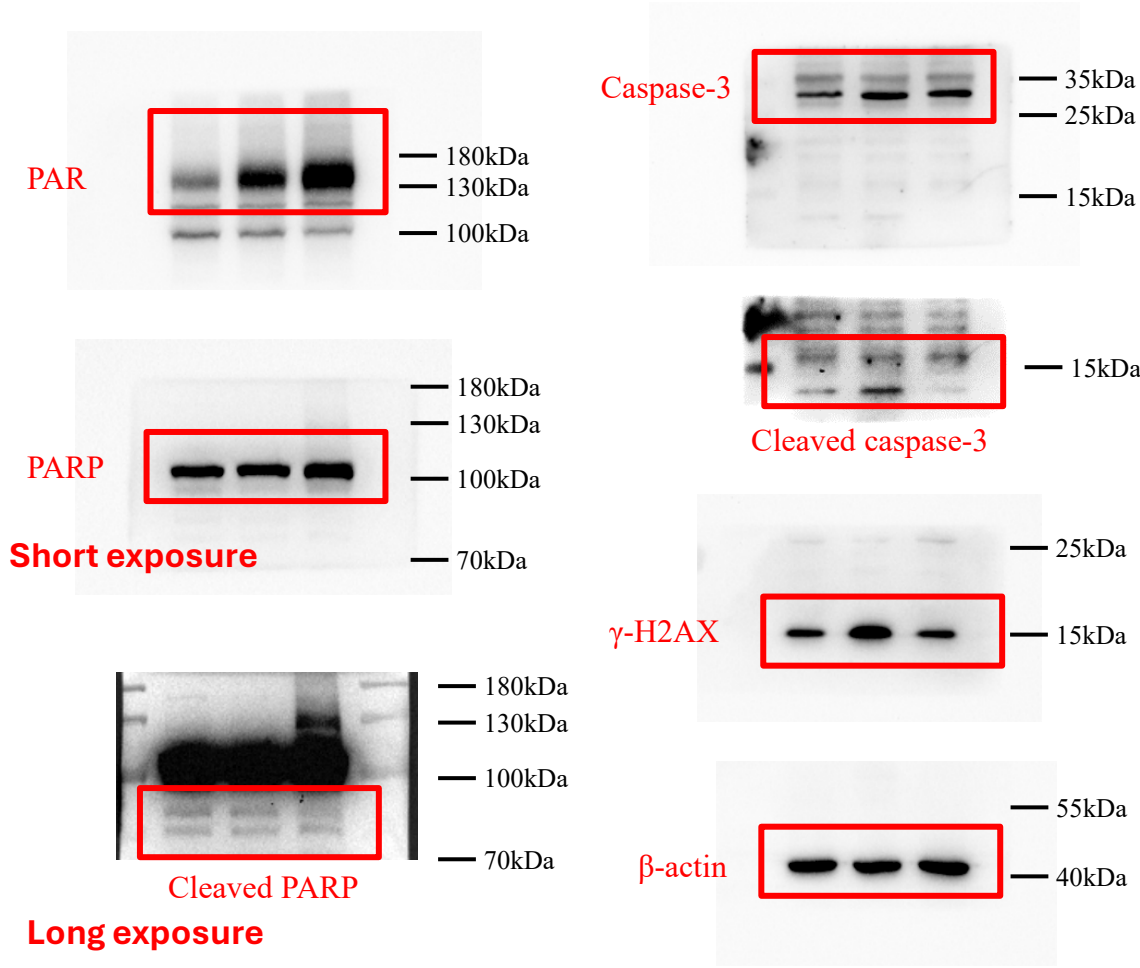

Figure 5F

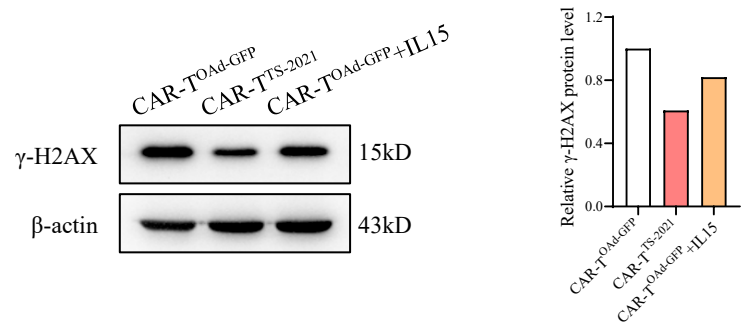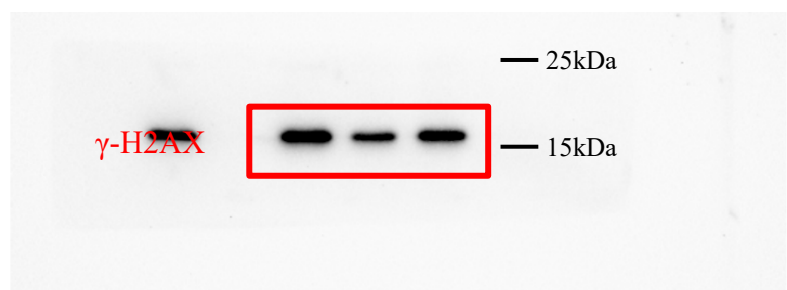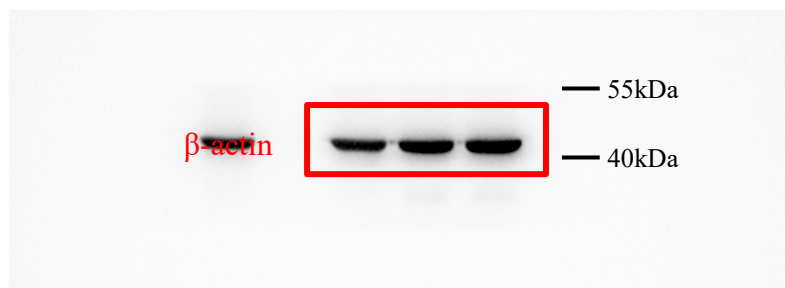

Figure 6B

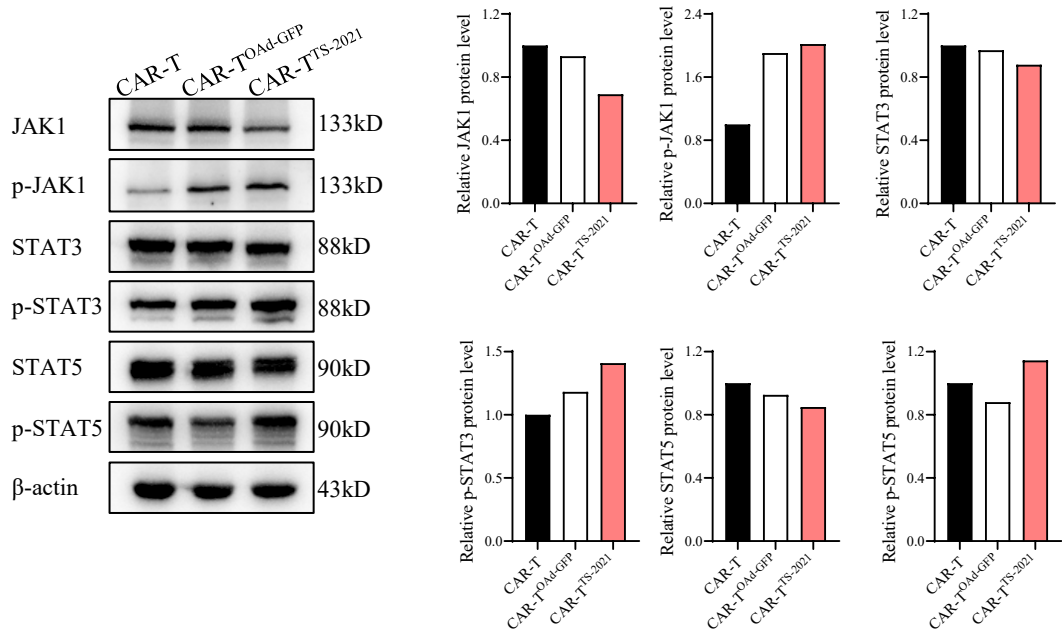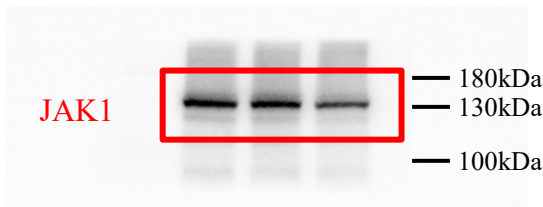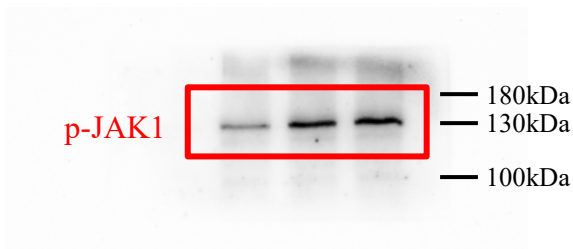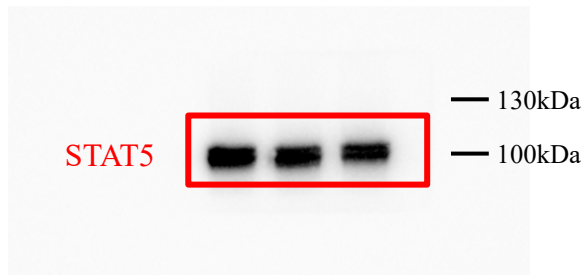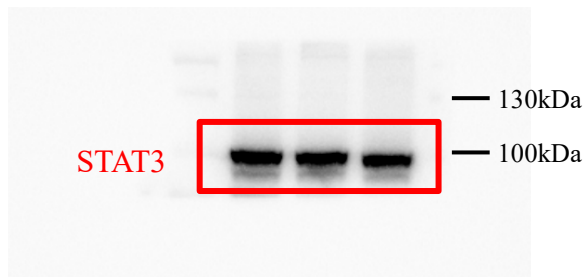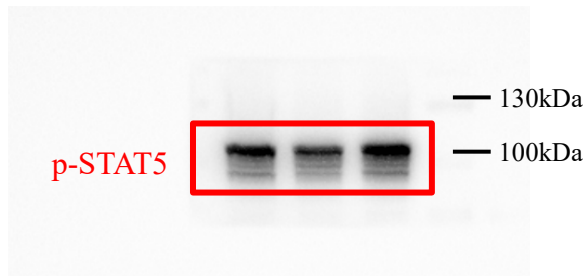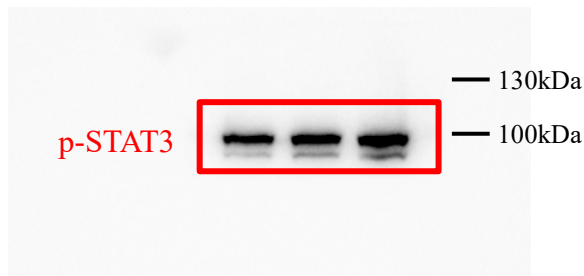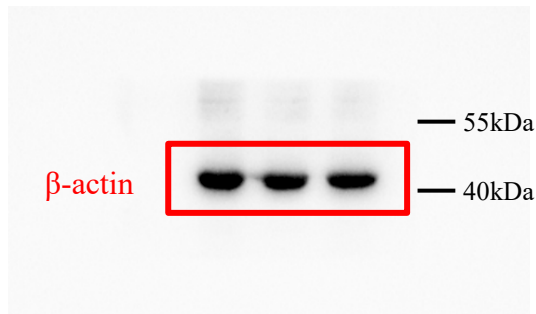

Figure 6B

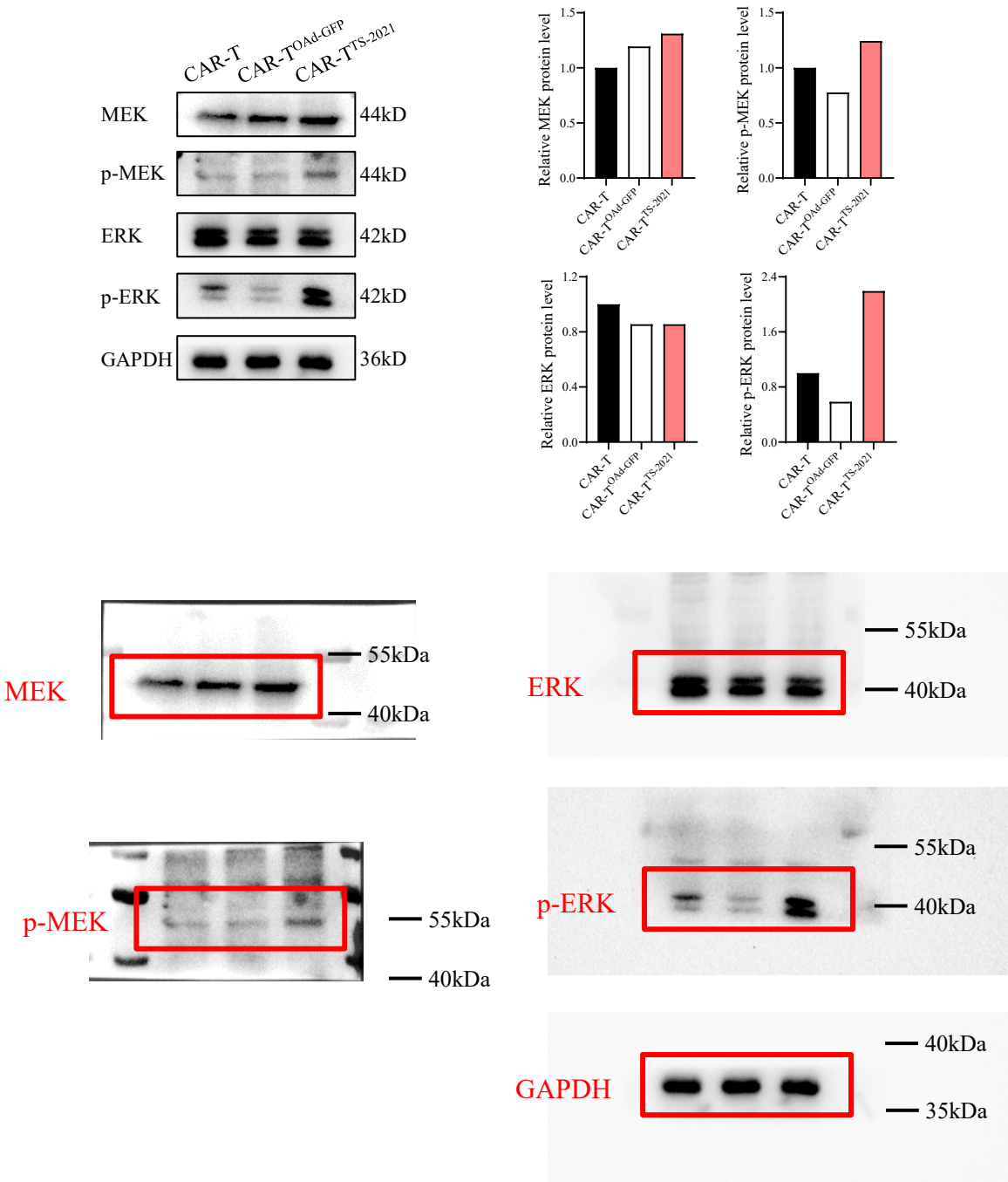

Figure 6E

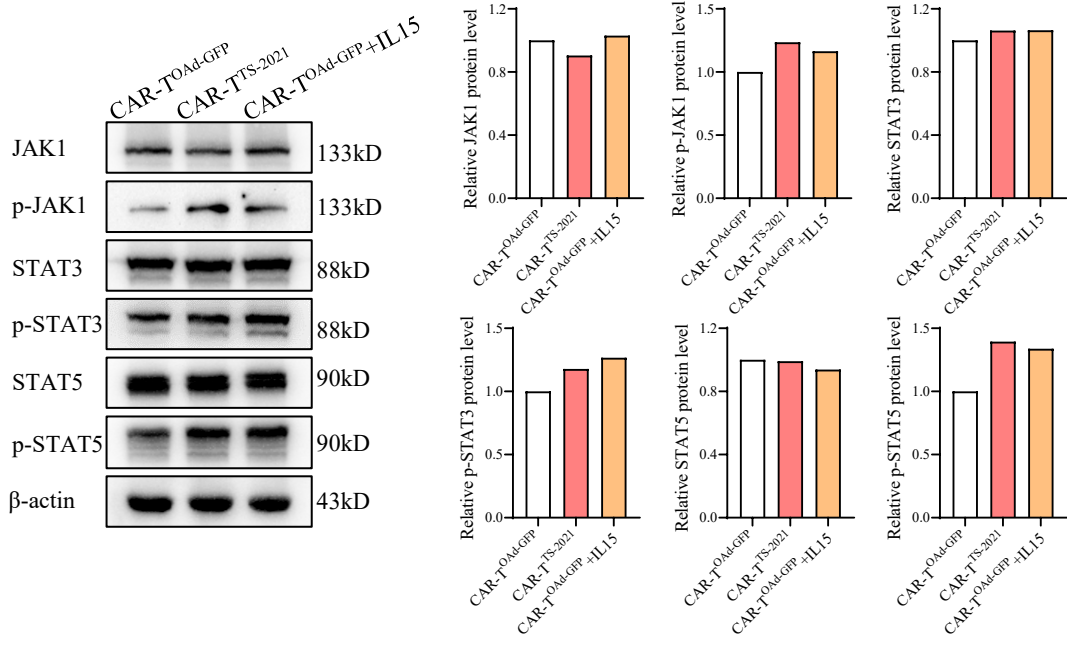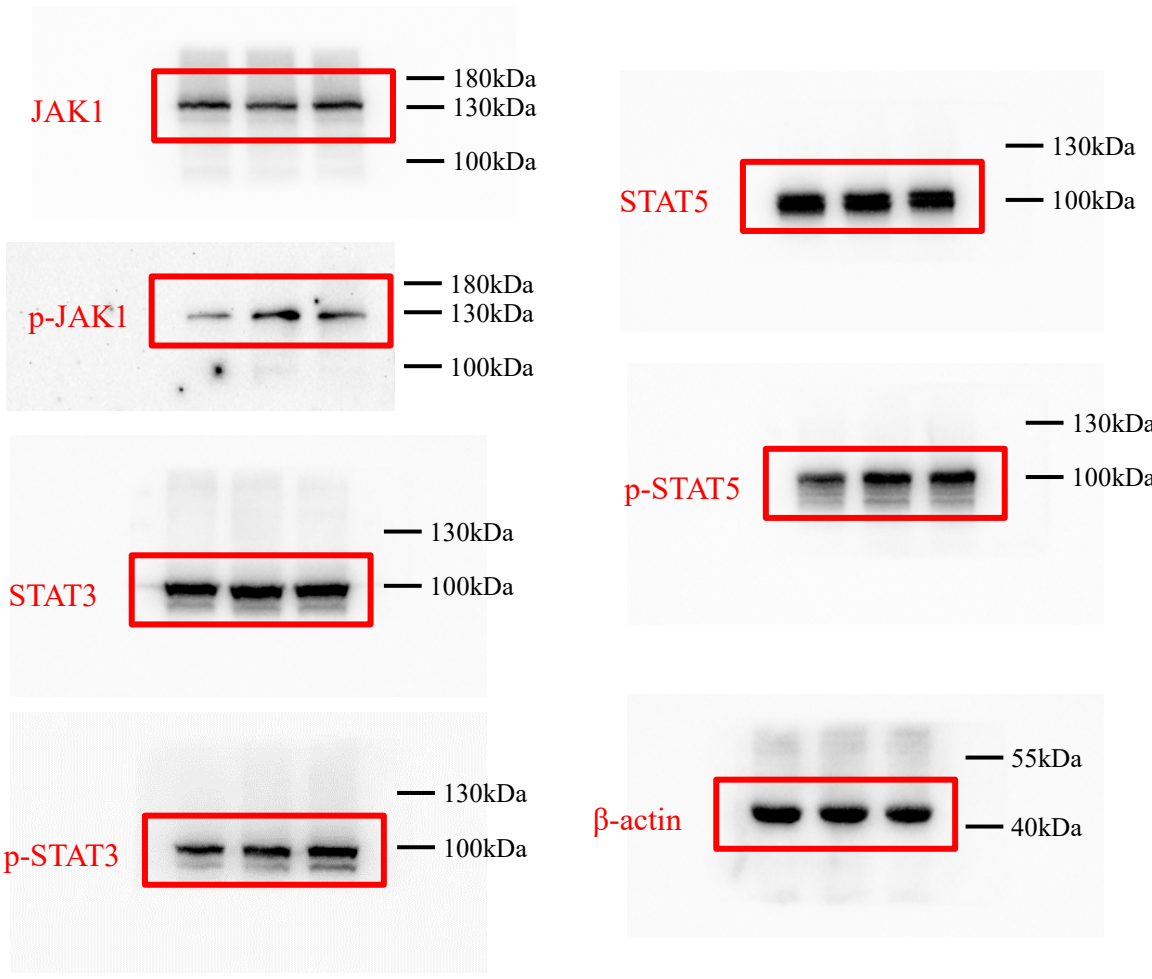

Figure 6E

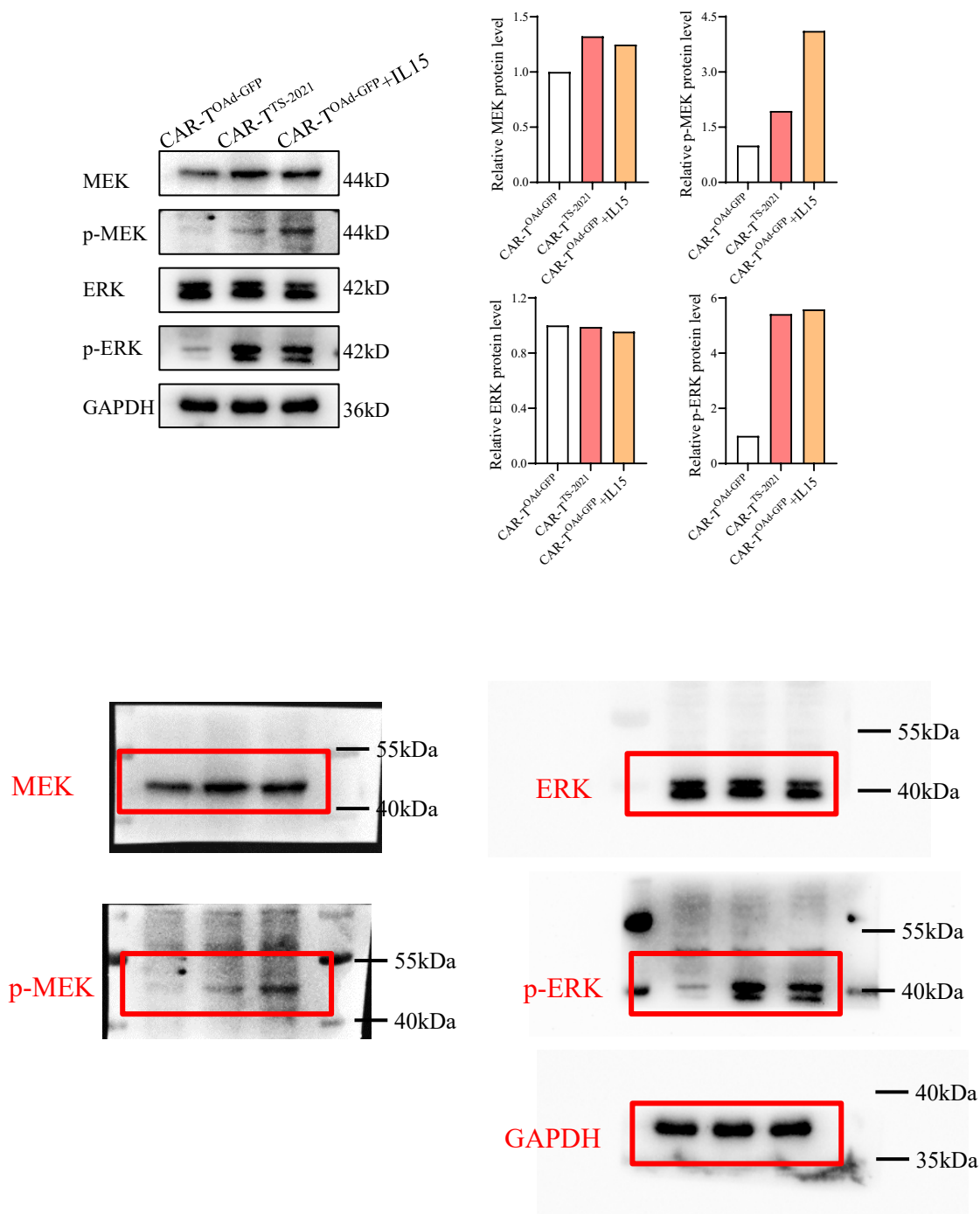

Supplement: Supplementary file 3 — Supplementary Material 3 [file 13046_2025_3431_MOESM3_ESM.pdf]
